# Compliance to Viral Load Monitoring Schedules Among Women Attending Prevention of Vertical HIV Transmission Services Before and During the COVID-19 Pandemic in Ehlanzeni District, Mpumalanga, South Africa

**DOI:** 10.1007/s10461-023-04192-z

**Published:** 2023-10-13

**Authors:** Thandiwe Elsie Mbira, Tendesayi Kufa, Gayle Gillian Sherman, Nobubelo Kwanele Ngandu, the PHANGISA Study Team

**Affiliations:** 1 HIV and Other Infectious Diseases Research Unit, South African Medical Research Council, Cape Town, South Africa; 2 Division of Epidemiology and Biostatistics, School of Public Health, University of the Witwatersrand, Johannesburg, South Africa; 3 Centre for HIV and STIs, National Institute for Communicable Diseases, Johannesburg, South Africa; 4 Department of Paediatrics and Child Health, Faculty of Health Sciences, University of the Witwatersrand, Johannesburg, South Africa

**Keywords:** Human immunodeficiency virus (HIV), Prevention of vertical HIV transmission (PVT), National PVT-guidelines, Compliance to viral load testing, COVID-19 pandemic, Lockdown restrictions

## Abstract

Human immunodeficiency virus (HIV) viral load (VL) monitoring was likely interrupted during the Coronavirus disease 2019 (COVID-19) pandemic. We used routine data on repeat VL testing among 667 prevention of vertical HIV transmission (PVT) clients in Ehlanzeni district, to determine compliance to VL testing recommendations and associated factors during different time periods: pre-COVID-19, transition, and COVID-19. Descriptive and multivariable Poisson regression analyses were conducted, with and without including revised PVT-guidelines rolled out in January-2020. Among 405 women with ≥ 2 VL tests, the overall median age was 30 years (interquartile range: 26–35 years). Compliance to recommended VL testing guidelines ranged between 81.5% (172/211) and 92.3% (191/207) at different time periods. Across all three periods and when revised PVT-guidelines were used, being compliant was significantly reduced among those with earliest VL = 50–999 copies/ml (incidence rate ratio (IRR) = 0.71 [95% confidence interval (CI) 0.61–0.82], p value < 0.001) and VL ≥ 1000 copies/ml (IRR = 0.18 [95% CI 0.09–0.36], p value < 0.001). When guideline revisions were excluded, compliance was only significantly reduced among those with VL ≥ 1000 copies/ml (IRR = 0.14 [95% CI 0.06–0.32], p value < 0.001) and increased during the COVID-19 period versus pre-COVID-19 (IRR = 1.10 [95% CI 1.05–1.15], p value < 0.001). Similar significant associations between compliance and VL level were observed when the COVID-19 period was analyzed separately. Significantly increased compliance to VL testing among the 25–34 years age-group versus younger women was also observed across all periods. These results highlight the importance of strengthening strategies such as short message service reminders and educational messaging, reaching all age-groups, to fast-track implementation targets for VL monitoring.

**Supplementary Information:**

The online version contains supplementary material available at 10.1007/s10461-023-04192-z.

## Introduction

The World Health Organization (WHO) recommended viral load (VL) testing for monitoring the effectiveness of antiretroviral treatment (ART) in human immunodeficiency virus (HIV) positive persons in 2013 [1]. VL monitoring is vital to tracking individual responses to ART, population-level effectiveness of ART, and progress towards United Nations Acquired Immunodeficiency Syndrome (UNAIDS) global targets for combating the HIV epidemic [2]. The UNAIDS’s third target in its 95-95-95 goals is to ensure that 95% of people on ART are virally suppressed by 2030 [3, 4]. However, progress towards the third target has been slow. In 2022, about 71% of people with HIV (PWHIV) globally were virally suppressed [5]. New interim targets for 2025 were developed to accelerate progress towards the 2030 goals. One of the main 2025 targets is to have 95% of pregnant and breastfeeding women with HIV (PBWHIV) having suppressed VL for elimination of vertical HIV transmission (EVT) [5–10]. The 2015 South African guidelines for Prevention of vertical HIV transmission (PVT) recommended a repeat VL in 1 month if VL ≥ 1000 copies/ml and within 6 months when VL < 1000 copies/ml, while the 2019 guidelines reduced the window period for repeat VL testing when VL = 50–999 copies/ml to 8–10 weeks and recommended the use of PVT codes on the National Health Laboratory Service (NHLS) VL requisition forms to electronically identify and permit frequent VL performed in PBWHIV at antenatal care (ANC), delivery, and postpartum, for rapid follow-up and care [11, 12].

Progress towards EVT has been slow and new infections continue to occur due to existing gaps in PVT; (i) new HIV infections during pregnancy, (ii) late HIV diagnosis and initiation of ART, and (iii) sub-optimal retention in care and viral suppression [13–21]. VL monitoring in this population has been sub-optimal, and compliance to repeat VL testing guidelines is not documented in South Africa [22–24]. Documenting compliance to VL testing in low-middle income countries is not yet common practice, yet it is crucial for understanding progress towards EVT. One example in Kenya reported that between 2016 and 2018 only 6% of PBWHIV who used public hospitals achieved a repeat VL test within the recommended schedule [25]. Additionally, PVT program activities are expected to have been impacted by the Coronavirus disease 2019 (COVID-19) pandemic and the restrictions on mobility and trade that were imposed to curb it.

In many countries, HIV services were interrupted, resources shifted towards the pandemic, and healthcare workers were allocated to COVID-19 tasks. Additionally, lockdown restrictions and fear of contracting COVID-19 made it difficult for people including pregnant and breastfeeding women to access healthcare services, and conducting face-to-face encounters was near impossible [10, 26, 27]. This may explain the decline of 25% or more in testing for HIV in six countries (Jamaica, Nigeria, Rwanda, Mozambique, Cameroon, Sierra Leone, and Botswana) among pregnant women [10]. In the 2022 UNICEF report, pregnant women from the Republic of Moldova missed and delayed HIV-related laboratories tests during the COVID-19 pandemic [28]. Reports of delays in VL monitoring among PWHIV when clinics and laboratories were functioning sub-optimally during COVID-19 have also been noted [29, 30]. Considering the existing gaps and effects of COVID-19, VL monitoring for timely management of HIV infection among PBWHIV was likely to be negatively affected, increasing the potential for vertical transmission. For these reasons, we conducted an exploratory study aimed at determining compliance (i.e., timely tests within guideline recommended number of weeks/months) to VL testing guidelines, and associated factors among PVT clients, before and during the COVID-19 pandemic in Ehlanzeni District, South Africa, within a context of changing PVT-guidelines.

## Methods

### Study Design and Setting

This was a retrospective cohort analysis of pregnant women and postpartum mothers, who were previously included in a facility-based cross-sectional study (the PHANGISA study). The PHANGISA study took place in the eight largest community healthcare centers of the Ehlanzeni District, located in rural Mpumalanga province of South Africa, from September to December 2019. Ehlanzeni’s HIV prevalence during pregnancy is the highest in the province and is one of the districts with the highest HIV prevalence (20%) in the general population nationally [31–33]. The PHANGISA study was designed to measure the prevalence of maternal VL non-suppression among pregnant or postpartum women with HIV and is described in detail in Ngandu et al. [34].

The 667 HIV-positive mothers who were enrolled in the PHANGISA study were retrospectively and virtually followed up using routine VL testing data from the NHLS’ Corporate Data Warehouse (NHLS-DW). Study participant names, surnames and dates of birth were used to link study participants with their respective HIV VL test results entered in the NHLS-DW from 1-April-2019 until 30-September-2021 (the study period). Once test results were linked on demographic data, the NHLS-DW unique identifier- created to assign multiple tests to a single person- was used to extract additional VL tests performed on the PHANGISA study participants within the study district and nationally.

### Sampling and Sample Size Determination

Sample precision was calculated assuming that all participants (N = 667) enrolled during the PHANGISA study would be included in the analysis. Based on this, the study had 100% power to detect assumed VL testing compliance of 38 ± 5% (based on the Mpumalanga province's known compliance) OR 90.2% power to detect compliance of 38 ± 3% OR 58.7% power to detect compliance of 38 ± 2% with 5% level of significance [35].

### Inclusion Criteria and Exclusion Criteria

The inclusion criteria for this current study were: all HIV-positive pregnant and postpartum mothers (i) aged 15 years and above enrolled in the primary study, (ii) with at least two VL test results in the NHLS-DW during the study period, and (iii) who reached 24 months postpartum only after the start of COVID-19 lockdown period (i.e., after 25-March-2020). Mothers with no NHLS-DW data or insufficient NHLS-DW data i.e., only one VL test throughout the study period, and those who reached 24 months postpartum before the COVID-19 period were excluded.

### Variables and Outcomes

#### Primary Outcome

**Fig. 1:**
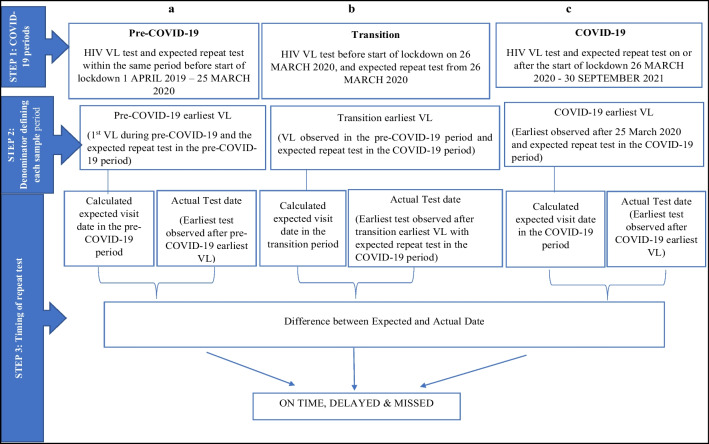
Steps followed to calculate compliance during pre-COVID-19, Transition, and COVID-19 periods, using the expected and actual VL tests. The expected repeat VL test was calculated as:—earliest VL test plus the number of weeks recommended by the guidelines before the next visit is due

The primary outcome measure was compliance to repeat VL testing within each period (i.e., pre-COVID-19, transition, or COVID-19), and was defined as the proportion of participants with a timely test (i.e., within guideline recommended number of weeks/months) out of the sample expected to have a repeat test during that period. The samples for each period were determined as follows: pre-COVID-19 sample (Fig. 1-a step-2)- included all participants with the earliest VL test (pre-COVID-19 earliest) and expected repeat VL test falling before the start of lockdown (i.e., before 25-March-2020); Transition sample (Fig. 1-b step-2)- included all participants with the latest observed VL test (transition earliest) in the pre-COVID-19 period and the expected repeat VL test falling during the COVID-19 period; COVID-19 period sample (Fig. 1-c step-2)- included all participants with the earliest observed VL test (COVID-19 earliest) and the expected repeat VL test both observed during the COVID-19 period.

The numerators for timing of repeat tests are defined in Fig. 1-step-3 and Table 1. The difference between the calculated expected date and the actual observed VL test date was used to determine the timing of the VL test (on-time, delayed or missed), within each of the three COVID-19 periods using 2015 and 2019 guidelines [10, 11].

**Table 1:** Calculation of compliance to repeat VL tests during COVID-19 periods using 2015 and 2019 South African PVT-guidelines

Guidelines	Previous VL	Expected time period to next visit as per the PVT-guidelines	Time window for ‘on-time’ VL test	Time window for ‘delayed’ VL test	Time window for ‘missed’ VL test
2015 guidelines	< 1000 copies/ml	6 months	6–9 months (i.e., plus half of 6)	> 9–12 months (i.e., until next due date)	> 12 months
	≥ 1000 copies/ml	4 weeks	4 weeks (unchanged)	5–8 weeks (i.e., until next due date)	> 8 weeks
2019 guidelines	< 50 copies/ml	6 months	6–9 months (i.e., plus half of 6)	> 9–12 months (i.e., until next due date)	> 12 months
	50–999 copies/ml	8 to 10 weeks	8–14 weeks (i.e., plus half of 8)	15–20 weeks (i.e., until next due date)	> 20 weeks
	≥ 1000 copies/ml	4 to 6 weeks	4–6 weeks (unchanged)	7–10 weeks (i.e., until next due date)	> 10 weeks

Two sets of calculations were done for the primary outcome: one accounted for the rollout of the revised 2019 guidelines from January-2020 onwards wherein the time to a repeat VL test when earliest VL = 50–999 copies/ml was shortened from 6-monthly to 8–10 weekly (referred to hereafter as the 2015/2019-PVT analysis); the other set assumed a potential delay in the rollout of the revised guidelines and thus used the 2015 recommendations throughout the study period (i.e., the 2015-PVT analysis).

#### Main Exposures

The first main exposure variable was the time periods: pre-COVID-19, transition, and COVID-19, defined by when VL tests were performed (Fig. 1). The second main exposure variable was the COVID-19 lockdown levels (1 through to 5) at the repeat VL test which changed differentially over the course of the COVID-19 period. This second exposure was only applied to the COVID-19 period.

#### Other Independent (Exposure) Variables

Sociodemographic factors collected during the PHANGISA study recruitment, perceived to influence healthcare uptake and HIV indicators were evaluated [34]. These included the participant’s PVT stage at recruitment (pregnant or postpartum), maternal age in years and further categorized (15–24, 25–34, 35–49 years) in secondary analysis to understand outcomes among the historically known HIV high-risk adolescent and young women age-group, level of education (ranging from no formal schooling to higher learning), marital status (never married or never lived with a partner, married or living together, widowed, divorced or separated), source of income (employed, spouse/partner, depend on others and government grant/no income), household monthly income categorized according to the national poverty line household cut-off (≤ R3200 or > R3200) [36], body mass index (BMI) (obese, overweight, normal, underweight) [37], partner's HIV status (positive, negative, unknown), condom use frequency (never, sometimes, always), planned pregnancy (yes or no), gestation at first ANC visit (0–12 weeks, 13–20 weeks, 21–40 weeks) and number of ANC visits (0–4 visits, 5–12 visits) were categorized according to the World Health Organization four-visit focused ANC model [38], timing of HIV diagnosis and ART initiation (before recent pregnancy, before 28 weeks gestation, after 28 weeks gestation, around labour/delivery, at postnatal before or after 6 months), current ART regimen (first-, second-, third- line, unknown), missed an ART dose last 7 days (yes or no), and facing any ART adherence challenges (yes or no).

### Data Management and Analysis

The primary outcome variable and exposure variables were generated using definitions explained earlier. Data were analyzed in Stata/BE-basic edition 17.0 [StataCorp, College Station, Texas USA] and R version 4.2.0 [2022-04-22 ucrt]. The socio-demographic data and the longitudinal VL tests data were merged. Data were cleaned to exclude participants not meeting the inclusion criteria for this analysis. Proportions were used to describe population characteristics and to report compliance to repeat VL testing. The outcome variable was also presented as a binary of on-time versus delayed or missed for the rest of the analyses. The distribution of the outcome by various exposures was assessed using a Chi-square test, for the pre-COVID-19, transition, and COVID-19 periods, separately. A partially overlapping samples z-test which compares proportions for two dichotomous samples was used to compare compliance to repeat VL testing between pre-COVID-19 and transition periods, pre-COVID-19 and COVID-19 periods, and transition and COVID-19 periods. Data in each comparison comprised a combination of paired (within patient) and unpaired observations [39]. Univariable and multivariable Poisson regression (with robust error variance) analyses were conducted to identify exposure factors associated with compliance to repeat VL testing across the three periods (including all repeat VL tests) and during the COVID-19 period separately (sub-analysis). Exposure variables with an overall univariable Poisson p value < 0.200 were selected for each multivariable model. Factors- age, education level, and income were kept in both overall multivariable analysis and sub-analysis a priori. A significance level of 0.050 was used in the multivariable analysis to indicate a significant association with compliance to VL testing. All descriptive and inferential statistics involving the outcome variable were conducted twice, i.e., for the 2015/2019-PVT analysis and the 2015-PVT analysis.

### Ethical Considerations

Ethics approval for the study was obtained from the South African Medical Research Council (SAMRC) ethics committee (EC021-6/2020). All PHANGISA participants gave consent for their routine data to be accessed and used for other related research in the future. Permission from the NHLS-DW gatekeeper was obtained to provide VL results matched to PHANGISA study participants.

## Results

### Sample Size Realization

**Fig. 2:**
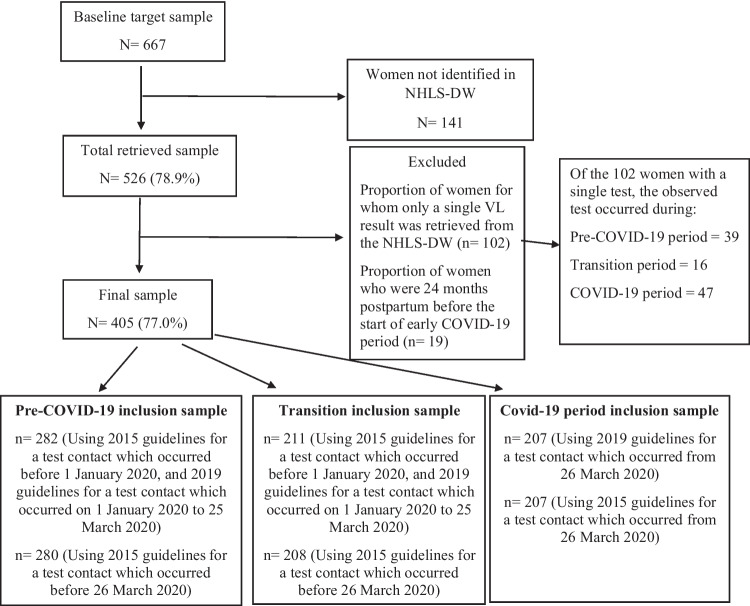
Final sample realization after exclusion and denominator for each period

Out of the target sample of 667, data for 526 (78.9%) participants were successfully extracted from the NHLS-DW using personal identifiers. A further 23% (121/526) of participants were excluded from the study using the study exclusion criteria, resulting in a final sample of 405 participants (Fig. 2). There were insufficient VL data for 243/667 (36.4%) participants. With the achieved final sample size (n = 405), the study had 89% power to detect VL testing compliance of 86 ± 3% with 5% level of significance. The number of participants included in the pre-COVID-19, transition, and COVID-19 exposure samples were 282, 211, and 207, respectively in the 2015/2019-PVT analysis and 280, 208, and 207, respectively, in the 2015-PVT analysis.

### Population Characteristics

#### Socio-demographics

Of the 405 included in the analysis, the overall median age of study participants was 30 years (interquartile range: 26–35) (Table 2). Over 90% of participants had at least grade 8 education level. About 60% of the participants were receiving less than R3200 of income monthly and approximately 35% did not know the HIV status of their male partner.

**Table 2:** characteristics of the PBWHIV included in the study (N = 405)

Variables	Categories	N	%
Maternal age (years)	Median (IQR)		30 (26.0–35.0)
	15–24 years	65	16.1
	25–34 years	234	57.8
	35–46 years	106	26.2
BMI	Median (IQR)		27 (23.4–32.0)
	Obese	141	34.8
	Overweight	115	28.4
	Normal	130	32.1
	Underweight	17	4.2
	Missing	2	0.5
Level of education	No formal school	2	0.5
	Grade 1–7	26	6.4
	Grade 8–12	231	57.0
	Certificate with grade12	103	25.4
	Diploma with grade12	41	10.1
	Postgrad certificate/diploma/honours degree	1	0.3
	Masters or PhD	1	0.3
Marital status	Never married/not cohabiting	239	59.0
	Married/cohabiting	153	37.8
	Widowed	4	1.0
	Divorced/separated	8	2.0
	Other	1	0.3
Source of income	Employed	119	29.4
	Spouse/partner	115	28.4
	Depend on others	61	15.1
	Government grant/no income	110	27.2
Monthly income	No income- R3200	235	58.0
	> R3200	170	42.0
Partner's HIV status	Positive	195	48.2
	Negative	69	17.0
	Unknown	141	34.8
Condom use frequency	Never	37	9.1
	Sometimes	156	38.5
	Always	210	51.9
	Missing	2	0.5
Planned pregnancy	No	217	53.6
	Yes	188	46.4

N= number of cases per category; *BMI* Body mass index; *IQR* Interquartile range

#### PVT-Related Factors

A majority (70%) of the participants were in the postpartum period at recruitment into the PHANGISA study (Table 3). Most (63.2%) attended their first ANC visit within 0–12 weeks of pregnancy, and around 75% were diagnosed with HIV before their current or recent pregnancy and had initiated ART.

**Table 3:** PVT-related factors

Variable	Categories	N	%
PVT stages	Pregnant	121	30.0
	Postpartum	284	70.0
Gestation at first ANC visit	0–12 weeks	256	63.2
	13–20 weeks	112	27.7
	21–40 weeks	37	9.1
Number of ANC visits	0–4 visits	145	35.8
	5–12 visits	260	64.2
HIV diagnosis	Before current/ recent pregnancy	304	75.1
	Before 28 weeks gestation	88	21.7
	After 28 weeks gestation	10	2,5
	Around labour/delivery	1	0.3
	At Postpartum before 6mnths	1	0.3
	At Postpartum after 6mnths	1	0.3
ART initiation	Before current/recent pregnancy	303	74.8
	Before 28 weeks gestation	85	21.0
	After 28 weeks gestation	8	2.0
	Around labour/delivery	1	0.3
	At Postpartum before 6mnths	3	0.7
	At Postpartum after 6mnths	4	1.0
	Missing	1	0.3
Current ART regimen	First line	332	82.0
	2nd/3rd line/unknown	71	17.5
	Missing	2	0.5
Missed an ART dose last 7 days	No	386	95.3
	Yes	19	4.7
Facing any ART adherence challenges	No	131	32.4
	Yes	274	67.7

n = number of cases per category; *ART* Antiretroviral treatment; *PVT* Prevention of vertical HIV transmission; *ANC* antenatal care

### Compliance to Repeat VL Testing During Pre-COVID-19, Transition, and COVID-19 Periods

**Fig. 3:**
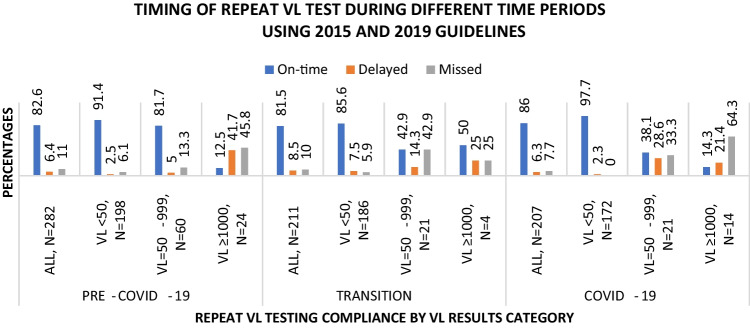
Compliance to repeat VL test during pre-COVID-19, transition, and COVID-19 periods, overall and by earliest VL (2015/2019-PVT analysis)

**Fig. 4:**
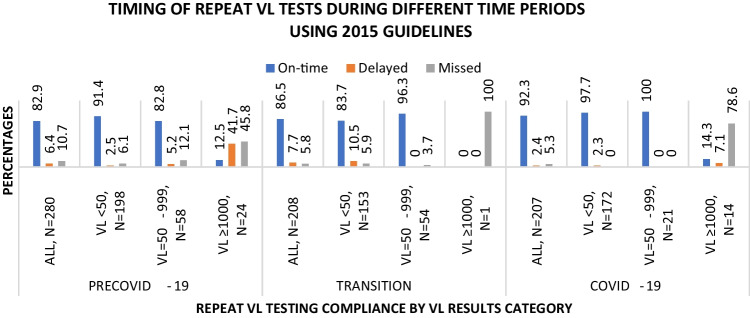
Compliance to repeat VL test during pre-COVID-19, transition, and COVID-19 periods, overall and by earliest VL (2015-PVT analysis)

Compliance to VL testing guidelines during pre-COVID-19, transition, and COVID-19 was determined, overall and by earliest VL status (Fig. 3, 2015/2019-PVT analysis). During the pre-COVID-19 period the proportion of women who were compliant to the VL test schedule, i.e., on time, was 82.6% (233/282). Compliance was 81.5% (172/211) during transition and 86.0% (178/207) during COVID-19. Compliance to repeat VL testing was compared between two periods at a time and there was no significant difference (all z-test p values > 0.05) between periods. In contrast, the 2015-PVT analysis (Fig. 4), a significant difference was observed between the pre-COVID-19 [82.9% (232/280)] and the COVID-19 [92.3% (191/207)] periods (difference in proportion: 9.4% [95% confidence interval (CI) 3.3–15.5], z-test p value < 0.001).

### Compliance to Repeat VL Testing by Exposure Variables During Pre-COVID-19, Transition, and COVID-19 Periods

Table S1(2015/2019-PVT analysis) and S2(2015-PVT analysis) show the distribution of compliance to repeat VL testing by baseline socio-demographic (S1A/S2A) and PVT-related (S1B/S2B) factors during different periods. During pre-COVID-19 compliance to the recommended VL testing schedule was higher—among older age groups, ≥ 25 years (≥ 81.3%), compared to the younger group, 15–24 years (2015/2019-PVT analysis: 68.2%, chi squared p value = 0.012) or (2015-PVT analysis: 67.4%, chi squared p value = 0.007). In all three time periods (pre-COVID-19, transition, and COVID-19), compliance was higher when earliest VL < 50 versus VL ≥ 1000 copies/ml, irrespective of PVT guidelines used (91.4% versus 12.5%, chi squared p values < 0.001, 86.6% (83.7% 2015-PVT analysis) versus 50.0% (0.0% 2015-PVT analysis), chi squared p value < 0.001 (chi squared p value = 0.003 2015-PVT analysis), and 97.7% versus 14.3%, chi squared p values < 0.001, respectively). However, compliance varied when earliest VL = 50–999 copies/ml. In the 2015/2019-PVT analysis, compliance when earliest VL = 50–999 versus VL ≥ 1000 copies/ml was higher only during pre-COVID-19 (S1B, ~ 81.7% versus ~ 12.5%), yet in the 2015-PVT analysis it was higher in all three periods (S2B, ~ 82.8–100.0% versus 0–14.3%). Compliance to VL testing also appeared to be lower among underweight participants during COVID-19 (S1A) and those on first line ART regimen during transition period (S1B), but only in the 2015/2019-PVT analysis.

### Factors Associated with Compliance to Repeat VL Testing

In both the 2015 and 2019-PVT analyses, compliance to VL testing was significantly reduced among those with earliest VL = 50–999 copies/ml or VL ≥ 1000 copies/ml versus VL < 50 copies/ml, irrespective of the time-period (VL = 50–999 Incidence Rate Ratio (IRR) = 0.70 [95% CI 0.61–0.81], Poisson p value < 0.001 and VL ≥ 1000 IRR = 0.18 [95% CI 0.09–0.36], Poisson p value < 0.001 across all three periods—Table 4; and VL = 50–999 IRR = 0.40 [95% CI 0.23–0.67], Poisson p value = 0.001 and VL ≥ 1000 IRR = 0.14 [95% CI 0.04–0.51], Poisson p value = 0.003 during the COVID-19 period—Table 5).

**Table 4:** Factors associated with compliance to VL testing 2015 or 2019 guidelines among PVT clients across the three time periods (N = 700 repeat tests)

	Number with on-time repeat VLs (row %)	Unadjusted		Adjusted	
		IRR (95% CI)	Poisson p value	IRR (95% CI)	Poisson p value
COVID-19 stages			0.348		
Pre-COVID-19	233 (82.6)	Ref			
Transition	172 (81.5)	0.99 [0.1, 1.07]	0.752		
COVID-19	178 (86.0)	1.04 [0.96, 1.12]	0.308		
Maternal age (years)	583 (83.3)	1.00 [1.00, 1.01]	0.241	1.00 [1.00, 1.01]	0.338
BMI groups			0.404		
Obese	2111 (85.4)	Ref			
Overweight	160 (81.2)	0.95 [0.87, 1.03]	0.243		
Normal	182 (83.1)	0.97 [0.90, 1.05]	0.494		
Underweight	29 (80.6)	0.94 [0.80, 1.12]	0.495		
Level of education			**0.097**		
None-grade 7	34 (77.3)	Ref		Ref	
Grade 8–12	333 (82.2)	1. 06 [0.90,1.26]	0.465	0.99 [0.86, 1.15]	0.945
Over high school	216 (86.1)	1.11 [0.94, 1.32]	0.209	1.04 [0.89, 1.20]	0.648
Marital status			0.432		
Never married/ not cohabiting	335 (82.1)	Ref			
Married/cohabiting	231 (85.2)	1.04 [0.97, 1.11]	0.275		
Widowed/divorced/separated	17 (81.0)	0.99 [0.80, 1.22]	0.896		
Source of income			0.677		
Employed	159 (82.4)	Ref		Ref	
Spouse/partner	168 (83.2)	1.01 [0.92, 1.10]	0.837	0.98 [0.91, 1.06]	0.664
Depend on others	90 (84.1)	1.02 [0.92, 1.13]	0.699	1.04 [0.95, 1.14]	0.432
Government grant/no income	166 (83.8)	1.02 [0.93, 1.11]	0.701	1.02 [0.94, 1.10]	0.661
Monthly income			0.292		
> R3200	228 (81.4)	Ref		Ref	
No income- R3200	355 (84.5)	1.04 [0.97, 1.11]	0.292	1.05 [0.98, 1.12]	0.146
Partner’s HIV status			0.247		
Positive	277 (81.7)	Ref			
Negative	103 (83.7)	1.02 [0.93, 1.12]	0.604		
Unknown	203 (85.2)	1.04 [0.97, 1.12]	0.249		
Condom use frequency*			0.919		
Never	48 (82.8)	Ref			
Sometimes	234 (83.9)	1.01 [0.89, 1.15]	0.838		
Always	300 (83.1)	1.00 [0.88, 1.14]	0.949		
Planned pregnancy			0.975		
No	308 (83.2)	Ref			
Yes	275 (83.3)	1.00 [0.94, 1.07]	0.975		
PVT stages			0.894		
Pregnant	178 (83.6)	Ref			
Postpartum	405 (83.2)	1.00 [0.93, 1.07]	0.894		
Baseline or previous VL			** < 0.001**		
VL < 50 copies/ml	510 (91.7)	Ref		Ref	
VL = 50–999 copies/ml	66 (64.7)	0.78 [0.61, 0.82]	0.000	0.70 [0.61, 0.81]	** < 0.001**
VL ≥ 1000 copies/ml	7 (16.7)	0.16 [0.09, 0.36]	0.000	0.18 [0.09, 0.36]	** < 0.001**
Gestation at first ANC visit			0.342		
0–12 weeks	370 (84.3)	Ref			
13–20 weeks	165 (82.1)	0.97 [0.90, 1.05]	0.498		
21–40 weeks	48 (80.0)	0.95 [0.83, 1.08]	0.442		
Number of ANC visits			0.301		
0–4 visits	209 (81.3)	Ref			
5–12 visits	374 (84.4)	1.04 [0.97, 1.11]	0.301		
ART initiation			0.548		
Before pregnancy	438 (83.0)	Ref			
During pregnancy, before 28 weeks	123 (83.7)	1.01 [0.93, 1.09]	0.835		
During pregnancy, after 28 weeks or during delivery or postnatal	22 (88.0)	1.06 [0.91, 1.23]	0.440		
Current ART regimen*			0.445		
First line	479 (82.9)	Ref			
2nd/3rd line/unknown	101 (85.6)	1.03 [0.95, 1.12]	0.445		
Missed an ART dose last 7 days			0.398		
No	560 (83.6)	Ref			
Yes	23 (76.7)	0.92 [0.75, 1.12]	0.398		
Facing any ART adherence challenges			0.819		
No	183 (82.8)	Ref			
Yes	400 (83.5)	1.01 [0.94, 1.08]	0.819		

*Variables with missing data, Factors with overall univariate p-value < 0.2 bolded, Bolded multivariate significant p-value < 0.05. *IRR* Incidence rate ratio; *CI* Confidence Interval; n = number of cases per category; *BMI* Body mass index; *ART* Antiretroviral treatment; *ANC* Antenatal Care; *PVT* Prevention of vertical HIV transmission

**Table 5:** Factors associated with compliance to VL testing 2015 or 2019 guidelines among PVT clients during COVID-19 period (N = 207)

	Number with on-time repeat VLs (row%)	Unadjusted		Adjusted	
		IRR (95% CI)	Poisson p value	IRR (95% CI)	Poisson p value
Maternal age (years)	178 (86.0)	1.01 [1.00, 1.02]	**0.126**	1.00 [1.00, 1.01]	0.203
BMI groups			0.995		
Obese	64 (86.5)	Ref			
Overweight	44 (80.0)	0.93 [0.79, 1.09]	0.340		
Normal	62 (93.9)	1.09 [0.98, 1.21]	0.133		
Underweight	7 (63.6)	0.74 [0.47, 1.16]	0.188		
Level of education			0.851		
None-grade 7	10 (83.3)	Ref		Ref	
Grade 8–12	105 (86.1)	1.03 [0.79, 1.34]	0.810	1.03 [0.80, 1.32]	0.844
Over high school	63 (86.3)	1.04 [0.79, 1.36]	0.799	1.03 [0.80, 1.32]	0.826
Marital status			0.570		
Never married/ not cohabiting	97 (84.4)	Ref			
Married/cohabiting	77 (88.5)	1.05 [0.94, 1.17]	0.389		
Widowed/divorced/separated	4 (80.0)	0.95 [0.61, 1.48]	0.816		
Source of income			0.834		
Employed	50 (84.8)	Ref		Ref	
Spouse/partner	55 (87.3)	1.03 [0.89, 1.19]	0.686	1.04 [0.94, 1.14]	0.433
Depend on others	26 (83.9)	0.99 [0.82, 1.20]	0.914	1.01 [0.91, 1.12]	0.812
Government grant/no income	47 (87.0)	1.03 [0.88, 1.19]	0.727	1.05 [0.94, 1.18]	0.355
Monthly income			0.970		
> R3200	73 (85.9)	Ref		Ref	
No income- R3200	105 (86.1)	1.00 [0.90, 1.12]	0.970	0.96 [0.87, 1.05]	0.334
Partner's HIV status			0.478		
Positive	89 (88.1)	Ref			
Negative	29 (82.9)	0.94 [0.80, 1.11]	0.471		
Unknown	90 (84.5)	0.96 [0.85, 1.08]	0.505		
Condom use frequency*			0.860		
Never	13 (86.7)	Ref			
Sometimes	70 (86.4)	1.00 [0.80, 1.24]	0.979		
Always	95 (85.6)	0.99 [0.80, 1.22]	0.908		
Planned pregnancy			0.341		
No	97 (88.2)	Ref			
Yes	81 (83.5)	0.95 [0.85, 1.06]	0.341		
Lockdown levels at repeat test			**0.173**		
Level 1	83 (88.3)	Ref			
Level 2	23 (92.0)	1.04 [0.91, 1.20]	0.558	1.02 [0.89, 1.18]	0.741
Level 3	58 (84.1)	0.95 [0.84, 1.08]	0.447	0.95 [0.89, 1.02]	0.166
Level 4	13 (72.2)	0.82 [0.61, 1.10]	0.184	0.80 [0.64, 1.00]	0.051
Level 5	1 (100.0)	1.13 [1.05, 1.11]	0.001	0.98 [0.92, 1.06]	0.665
PVT stages					
Pregnant	42 (82.4)	Ref			
Postpartum	136 (87.2)	1.06 [0.92, 1.22]	0.428		
Baseline or previous VL			** < 0.001**		
VL < 50 copies/ml	168 (97.7)	Ref		Ref	
VL = 50–999 copies/ml	8 (38.1)	0.39 [0.23, 0.67]	0.001	0.40 [0.23, 0.67]	**0.001**
VL ≥ 1000 copies/ml	2 (14.3)	0.15 [0.04, 0.53]	0.003	0.14 [0.04, 0.51]	**0.003**
Gestation at first ANC visit			0.888		
0–12 weeks	117 (85.4)	Ref			
13–20 weeks	45 (88.2)	1.03 [0.91, 1.17]	0.600		
21–40 weeks	16 (84.2)	0.99 [0.80, 1.21]	0.894		
Number of ANC visits			0.665		
0–4 visits	66 (84.6)	Ref			
5–12 visits	112 (86.8)	1.02 [0.91, 1.15]	0.665		
ART initiation			**0.207**		
Before pregnancy	128 (88.3)	Ref		Ref	
During pregnancy, before 28 weeks	43 (81.1)	0.92 [0.80, 1.06]	0.248	0.95 [0.86, 1.06]	0.378
During pregnancy, after 28 weeks or during delivery or postnatal	7 (77.8)	0.88 [0.62, 1.26]	0.485	0.96 [0.75, 1.23]	0.746
Current ART regimen*			0.794		
First line	150 (86.2)	Ref			
2nd/3rd line/unknown	27 (84.4)	0.98 [0.83, 1.15]	0.794		
Missed an ART dose last 7 days			0.638		
No	170 (86.3)	Ref			
Yes	8 (80.0)	0.93 [0.68, 1.27]	0.638		
Facing any ART adherence challenges			0.935		
No	60 (85.7)	Ref			
Yes	118 (86.1)	1.00 [0.89, 1.13]	0.935		

*Variables with missing data, Factors with overall univariate p-value < 0.2 bolded, Bolded multivariate significant p-value < 0.05. *IRR* Incidence rate ratio; *CI* Confidence Interval; n = number of cases per category; *BMI* Body mass index; *ART*, Antiretroviral treatment; *ANC* Antenatal Care; *PVT* Prevention of vertical HIV transmission

In the 2015-PVT analysis and irrespective of the time-period, the likelihood of being compliant was significantly reduced only among those with earliest VL ≥ 1000 copies/ml (versus VL < 50 copies/ml) (IRR = 0.14 [95% CI 0.06–0.32], Poisson p value < 0.001 across all periods—Table 6; and IRR = 0.15 [95% CI 0.04–0.52], Poisson p value = 0.003 during the COVID-19 period—Table 7). There was significant overall increased compliance during the COVID-19 versus pre-COVID-19 period IRR = 1.10 [95% CI 1.04–1.15], Poisson p value < 0.001, Table 6.

**Table 6:** Factors associated with compliance to VL testing 2015 guidelines among PVT clients across the three time periods (N = 695 repeat tests)

	Number with on-time repeat VLs (row %)	Unadjusted		Adjusted	
		IRR (95% CI)	Poisson p value	IRR (95% CI)	Poisson p value
COVID-19 stages			**0.002**		
Pre-COVID-19	232 (82.9)	Ref		Ref	
Transition	180 (86.5)	1.04 [0.97, 1.13]	0.260	0.97 [0.90, 1.04]	0.360
COVID-19	191 (92.3)	1.11 [1.04, 1.19]	0.001	1.10 [1.04, 1.15]	** < 0.001**
Maternal age (years)	603 (86.8)	1.00 [1.00, 1.01]	**0.128**	1.00 [1.00, 1.01]	0.390
BMI groups			0.477		
Obese	216 (88.2)	Ref			
Overweight	167 (86.1)	0.98 [0.91, 1.05]	0.521		
Normal	188 (85.8)	0.97 [0.91, 1.05]	0.460		
Underweight	31 (86.1)	0.98 [0.85, 1.12]	0.740		
Level of education			**0.100**		
None-grade 7	34 (77.3)	Ref		Ref	
Grade 8–12	349 (86.6)	1.12 [0.95, 1.32]	0.176	1.06 [0.92, 1.23]	0.432
Over high school	220 (88.7)	1.15 [0.97, 1.36]	0.104	1.08 [0.93, 1.26]	0.288
Marital status			0.974		
Never married/ not cohabiting	350 (86.4)	Ref			
Married/cohabiting	236 (87.7)	1.02 [0.96, 1.08]	0.617		
Widowed/divorced/separated	17 (81.0)	0.94 [0.76, 1.16]	0.544		
Source of income			0.604		
Employed	168 (87.5)	Ref		Ref	
Spouse/partner	174 (86.6)	0.99 [0.92, 1.07]	0.783	0.98 [0.92, 1.05]	0.552
Depend on others	94 (88.7)	1.01 [0.93, 1.11]	0.762	1.02 [0.94, 1.09]	0.678
Government grant/no income	167 (85.2)	0.97 [0.90, 1.05]	0.510	0.98 [0.91, 1.05]	0.529
Monthly income			0.259		
> R3200	237 (85.0)	Ref		Ref	
No income- R3200	366 (88.0)	1.04 [0.97, 1.10]	0.259	1.06 [1.00, 1.12]	0.063
Partner's HIV status			0.543		
Positive	288 (85.7)	Ref			
Negative	108 (88.5)	1.03 [0.96, 1.12]	0.414		
Unknown	207 (87.3)	1.02 [0.95, 1.09]	0.572		
Condom use frequency*			0.685		
Never	49 (86.0)	Ref			
Sometimes	244 (88.1)	1.02 [0.91, 1.15]	0.674		
Always	309 (86.1)	1.00 [0.89, 1.12]	0.983		
Planned pregnancy			0.972		
No	320 (86.7)	Ref			
Yes	283 (86.8)	1.00 [0.94, 1.06]	0.972		
PVT stages			0.655		
Pregnant	184 (87.6)	Ref			
Postpartum	419 (86.4)	0.99 [0.93, 1.05]	0.655		
Baseline or previous VL			** < 0.001**		
VL < 50 copies/ml	477 (91.2)	Ref		Ref	
VL = 50–999 copies/ml	121 (91.0)	1.00 [0.94, 1.06]	0.935	1.01 [0.95, 1.08]	0.658
VL ≥ 1000 copies/ml	5 (12.8)	0.14 [0.06, 0.33]	< 0.001	0.14 [0.06, 0.32]	** < 0.001**
Gestation at first ANC visit			**0.169**		
0–12 weeks	383 (88.1)	Ref		Ref	
13–20 weeks	171 (85.5)	0.97 [0.91, 1.03]	0.389	0.99 [0.93, 1.04]	0.615
21–40 weeks	49 (81.7)	0.93 [0.82, 1.05]	0.238	0.95 [0.85, 1.05]	0.329
Number of ANC visits			0.972		
0–4 visits	224 (86.8)	Ref			
5–12 visits	379 (86.7)	1.00 [0.94, 1.06]	0.972		
ART initiation			0.301		
Before pregnancy	451 (86.4)	Ref			
During pregnancy, before 28 weeks	128 (86.5)	1.00 [0.93, 1.08]	0.978		
During pregnancy, after 28 weeks or during delivery or postnatal	24 (96.0)	1.11 [1.02, 1.21]	0.018		
Current ART regimen*			0.466		
First line	497 (86.4)	Ref			
2nd/3rd line/unknown	103 (88.8)	1.03 [0.96, 1.10]	0.466		
Missed an ART dose last 7 days			0.612		
No	578 (86.9)	Ref			
Yes	25 (83.3)	0.96 [0.81, 1.13]	0.612		
Facing any ART adherence challenges			0.368		
No	187 (85.0)	Ref			
Yes	416 (87.6)	1.03 [0.97, 1.10]	0.368		

*Variables with missing data, Factors with overall univariate p-value < 0.2 bolded, Bolded multivariate significant p-value < 0.05. *IRR* Incidence rate ratio; *CI* Confidence Interval; n = number of cases per category; *BMI* Body mass index; *ART* Antiretroviral treatment; *ANC* Antenatal Care; *PVT* Prevention of vertical HIV transmission

**Table 7:** Factors associated with compliance to VL testing under 2015 guidelines among PVT clients during COVID-19 period (N = 207)

	Number with on-time repeat VLs (row%)	Unadjusted		Adjusted	
		IRR (95% CI)	Poisson p value	IRR (95% CI)	Poisson p value
Maternal age (years)	191 (92.3)	1.00 [1.00, 1.01]	0.398	1.00 [1.00, 1.00]	0.971
BMI groups			0.981		
Obese	69 (93.2)	Ref			
Overweight	48 (87.3)	0.94 [0.83, 1.05]	0.273		
Normal	64 (97.0)	1.04 [0.96, 1.12]	0.305		
Underweight	9 (81.8)	0.88 [0.66, 1.17]	0.370		
Level of education			0.483		
None-grade 7	10 (83.3)	Ref			
Grade 8–12	113 (92.6)	1.11 [0.86, 1.44]	0.423	1.07 [0.86, 1.33]	0.561
Over high school	68 (93.2)	1.12 [0.86, 1.45]	0.403	1.07 [0.87, 1.32]	0.494
Marital status			0.522		
Never married/ not cohabiting	107 (93.0)	Ref			
Married/cohabiting	80 (92.0)	0.99 [0.91, 1.07]	0.773		
Widowed/divorced/separated	4 (80.0)	0.86 [0.55, 1.34]	0.503		
Source of income			0.856		
Employed	55 (93.2)	Ref			
Spouse/partner	57 (90.5)	0.97 [0.87, 1.08]	0.580	0.98 [0.91, 1.07]	0.676
Depend on others	30 (96.8)	1.04 [0.94, 1.14]	0.437	0.99 [0.95, 1.04]	0.825
Government grant/no income	49 (90.7)	0.97 [0.87, 1.09]	0.630	0.98 [0.91, 1.06]	0.635
Monthly income			0.761		
> R3200	79 (92.9)	Ref			
No income- R3200	112 (91.8)	0.99 [0.91, 1.07]	0.761	0.97 [0.93, 1.02]	0.242
Partner’s HIV status			**0.169**		
Positive	95 (94.1)	Ref			
Negative	34 (97.1)	1.03 [0.96, 1.11]	0.401	1.00 [0.98, 1.03]	0.917
Unknown	62 (87.3)	0.93 [0.84, 1.03]	0.151	0.96 [0.88, 1.05]	0.357
Condom use frequency*			**0.022**		
Never	15 (100.0)	Ref			
Sometimes	77 (95.1)	0.95 [0.90, 1.00]	0.046	0.97 [0.92, 1.03]	0.256
Always	99 (89.2)	0.89 [0.84, 0.95]	0.001	0.98 [0.93, 1.03]	0.351
Planned pregnancy			**0.203**		
No	104 (94.6)	Ref			
Yes	87 (89.7)	0.95 [0.87, 1.03]	0.203	0.97 [0.93, 1.02]	0.255
Lockdown levels at repeat test			**0.248**		
Level 1	89 (94.7)	Ref			
Level 2	23 (92.0)	0.97 [0.86, 1.10]	0.654	1.03 [0.90, 1.19]	0.637
Level 3	62 (89.8)	0.95 [0.86, 1.04]	0.270	0.98 [0.94, 1.02]	0.369
Level 4	16 (88.9)	0.94 [0.79, 1.11]	0.468	0.89 [0.76, 1.06]	0.186
Level 5	1 (100.0)	1.06 [1.01, 1.11]	0.026	1.04 [0.97, 1.12]	0.230
PVT stages			0.558		
Pregnant	46 (90.2)	Ref			
Postpartum	145 (93.0)	1.03 [0.93, 1.14]	0.558		
Baseline or previous VL			** < 0.001**		
VL < 50 copies/ml	168 (97.7)	Ref			
VL = 50–999 copies/ml	21 (100.0)	1.02 [1.00, 1.05]	0.046	1.02 [0.99, 1.06]	0.189
VL ≥ 1000 copies/ml	2 (14.3)	0.15 [0.04, 0.53]	0.003	0.15 [0.04, 0.52]	**0.003**
Gestation at first ANC visit			0.681		
0–12 weeks	127 (92.7)	Ref			
13–20 weeks	47 (92.2)	0.99 [0.91, 1.09]	0.901		
21–40 weeks	17 (89.5)	0.97 [0.82, 1.13]	0.667		
Number of ANC visits			0.568		
0–4 visits	73 (93.6)	Ref			
5–12 visits	118 (91.5)	0.98 [0.90, 1.06]	0.568		
ART initiation			0.789		
Before pregnancy	135 (93.1)	Ref			
During pregnancy, before 28 weeks	47 (88.7)	0.95 [0.86, 1.06]	0.369		
During pregnancy, after 28 weeks or during delivery or postnatal	9 (100.0)	1.07 [1.03, 1.22]	0.002		
Current ART regimen*			**0.139**		
First line	159 (91.4)	Ref			
2nd/3rd line/unknown	31 (96.9)	1.06 [0.98, 1.15]	0.139	1.00 [0.95, 1.05]	0.913
Missed an ART dose last 7 days			0.808		
No	182 (92.4)	Ref			
Yes	9 (90.0)	0.97 [0.79, 1.20]	0.808		
Facing any ART adherence challenges			0.415		
No	63 (90.0)	Ref			
Yes	128 (93.4)	1.04 [0.95, 1.14]	0.415		

*Variables with missing data, Factors with overall univariate p-value < 0.2 bolded, Bolded multivariate significant p-value < 0.05. *IRR* Incidence rate ratio; *CI* Confidence Interval; n = number of cases per category; *BMI* Body mass index; *ART* Antiretroviral treatment; *ANC* Antenatal Care; *PVT* Prevention of vertical HIV transmission

When age was categorized to investigate the differences between the historically known high-risk age group (15–24 years) and older age groups, all results remained the same except for age, across the three stages and irrespective of PVT guidelines. There was significant overall increased compliance among the 25–34 years age-group versus younger (15–24 years) women (IRR = 1.12 [95% CI 1.01–1.23], Poisson p value = 0.027) in the 2015/2019-PVT analysis-Table S3; and (IRR = 1.10 [95% CI 1.00–1.20], Poisson p value = 0.035) in the 2015-PVT analysis-Table S4).

## Discussion

This study documented compliance to VL testing in PBWHIV before and during the COVID-19 pandemic in a rural district in South Africa, during April-2019 to September-2021. Given the study period overlapped with the rollout of the 2019-PVT guidelines in January-2020, three months before the COVID-19 hard lockdown in South Africa, we considered a scenario with and without the use of the revised PVT guidelines to delineate the effect of the pandemic in the context of changing guideline practices. Our results indicated that compliance to VL testing was largely affected by the frequency of recommended repeat tests irrespective of the COVID-19 pandemic status and that the COVID-19 pandemic lockdown delayed the implementation of the revised PVT-guidelines.

The increased likelihood of being compliant during the COVID-19 period compared to the pre-COVID-19 period, observed only in the 2015-PVT analyses where frequent visits for low-level viraemia were not applied, supports the delayed implementation of the revised guidelines. The higher proportion of delayed/missed VL testing visits among those on first-line ART regimen during the transition period when frequent visits were applied, also supports delayed implementation of the revised guidelines, given the majority of those with increasing VL would be expected to be on first-line regimen. This likely reflected the sub-group with low-level viraemia who might have been prescribed the 6-monthly repeat visit instead of the 8–10 weekly visit. The association between first-line ART regimen and high VL was also confirmed in the baseline cross-sectional point prevalence study, supporting a delay in treatment intervention for those with detectable viraemia [34]. These observations confirm that the COVID-19 pandemic interrupted the training of healthcare workers for the implementation of the 2019-PVT guidelines. Training should have been resumed with urgency after the pandemic restrictions were eased.

The second major finding of this study raises a conundrum over the implementation of the revised and previous guidelines alike. According to this study, compliance to more frequent VL testing visits, which are prescribed for those at highest risk of VHT, was sub-optimal even before the COVID-19 pandemic. In both PVT-guideline scenarios and across the entire study period, compliance to VL testing was significantly reduced among those with high-level viraemia VL ≥ 1000 copies/ml, who were routinely prescribed 4–6 weekly clinic visits. Compliance was significantly reduced when VL = 50–999 copies/ml (i.e., low-level viraemia), only when the revised guidelines were used, wherein low-level viraemia clients are prescribed 8–10 weekly clinic visits instead of the previous 6-monthly visits. Shortening time to repeat clinic visits for those with low-level viraemia was motivated by growing evidence of high risk for VHT [40]. The revised guidelines imply an increase in the population of those requiring frequent visits amid sub-optimal prior implementation. Implementation interventions need to be prioritized urgently.

To improve VL monitoring compliance, continuous evaluation and strengthening of the existing interventions, such as short message service (SMS) appointment reminders sent to PVT clients as part of Accelerating Programme Achievements to Control the Epidemic program is vital [41]. SMS reminders have previously been shown to improve retention and viral suppression among PVT clients, early infant diagnosis follow-up testing, and uptake of repeat HIV testing [42–44]. Recommendations for improving outcomes include (i) eLABS, that deliver VL results to healthcare workers digitally as soon as the results are ready and are piloting SMS reminders for patients [45] and (ii) Results for Action (RfA) reports, that collate HIV VL results weekly and identify high VL belonging to PBWHIV for clinical action, using codes introduced in the 2019-PVT guidelines [11, 12, 46, 47]. Furthermore, some previously proposed interventions that have shown potential to improve and encourage VL testing include educational efforts on the benefits of VL testing, using point-of-care VL testing for quick result turnaround times and enhanced access to VL testing in resource-limited settings, enhanced adherence counselling, compensating patients, and services aimed at young PBWHIV as they are less likely to receive VL testing [33, 48–51]. Periodic evaluation will also help in understanding the gaps that exist around implementation of guidelines and assist in addressing the impact of natural disasters such as COVID-19 pandemic on PWHIV.

The better compliance to VL testing observed among older compared to younger women, observed when age was categorized in a secondary analysis, is not unexpected, given known challenges in achieving viral suppression, uptake of care and VHT risk in younger women [34, 50–53]. The insignificance of age in the COVID-19 period sub-analyses, regardless of PVT-guidelines used, could be due to the universally enforced COVID-19 movement restrictions. Other participant sociodemographic factors such as education and income were not risk factors unlike in other related PVT indicators [50, 51, 54, 55].

*This study had limitations and strengths*. Although the message of delayed implementation of guidelines is undeniable, the observed levels of compliance to VL testing are best interpreted as an over-estimation. This study had a higher (~ 82%) compliance to repeat VL testing among PVT clients during pre-COVID-19 compared to what was reported for the general population during 2016 in South Africa (47.7–56.4%) [35], and what was seen among PVT clients in Kenya (only 6% of repeat VL testing within recommended schedule) [25] and Republic of Moldova during the COVID-19 pandemic among PWHIV (28.6%) [28], but comparable to observations of a similarly small PWHIV sub-population in southwestern Uganda (80.3%) [55]. A high proportion of women were excluded because of only one VL test observed (102/667) in the NHLS-DW, despite searching nation-wide routine data to account for women’s mobility. Deaths of study participants due to COVID-19 or loss-to-follow-up may have contributed to the exclusions. However, the realized study sample was highly powered for the analyses of repeated measures and sufficiently portrayed changes (or lack thereof) over time. In contrast, the sub-samples used for reporting point prevalence of compliance to VL testing at each time-period were insufficient for external validity. However, the distribution of age in the remaining sample is similar to that in the baseline sample as well as the national VL monitoring survey, indicating that the sample that could not be traced on the NHLS-DW (mostly due to inconsistent use of patient demographics and the absence of a unique health identifier) was random [22, 34]. This qualifies the appropriateness and reliability of the rest of the results attained from the repeated measures regression analyses.

The sociodemographic factors were collected at one time-point, thus the effect of time-changing factors was not measured.

Although there are a few limitations, the study managed to combine observations of routine VL testing among PBWHIV over a period of 30 months, including 12 months before the onset of the pandemic in South Africa, with an assessment of changes in PVT-guidelines within that period, and raised an alarm for implementation priorities. Given that there have been extremely limited assessments of compliance to VL testing for PBWHIV in the past, the implementation and evaluation of the 2019 recommendation to use PVT codes on the NHLS laboratory requisition forms to identify and track VL performed in PBWHIV at ANC, delivery and postpartum, needs to be prioritized.

## Conclusions

Although the point prevalence of compliance to repeat VL testing were over-estimated, the study had high power to accurately monitor VL testing over a 30-month period and delineate the effect of the COVID-19 pandemic within a context of rolling out revised PVT-guidelines. Two major observations were made: Compliance to repeat VL testing was significantly reduced among those with viraemia and requiring frequent visits, both before and during the COVID-19 pandemic; and the rollout of the revised PVT-guidelines for shortening time to the next clinic visit for a low-level viraemia, was delayed due to the COVID-19 pandemic restrictions initiated barely three months into the rollout. To improve compliance to repeat VL testing, especially among those with detectable VL and young women, there is a need to strengthen interventions by (i) training healthcare workers on the new guidelines, (ii) educational messaging on VL testing benefits, (iii) SMS appointment reminders, and (iv) adolescent-PBWHIV friendly services, all which have shown a potential to improve uptake and timeous linkage for appropriate treatment and care.

### Supplementary Information

Below is the link to the electronic supplementary material.

Supplementary file1 (DOCX 118 KB)
