# Supplementary material for: Compliance to Viral Load Monitoring Schedules Among Women Attending Prevention of Vertical HIV Transmission Services Before and During the COVID-19 Pandemic in Ehlanzeni District, Mpumalanga, South Africa
Source: AIDS Behav. 2023 Oct 13;28(3):868–85. doi: 10.1007/s10461-023-04192-z (PMC10896817; doi:10.1007/s10461-023-04192-z)
Supplement: Supplementary file 1 — Supplementary file1 (DOCX 118 KB) [file 10461_2023_4192_MOESM1_ESM.docx]

**Compliance to viral load monitoring schedules among women attending Prevention of Vertical HIV Transmission services before and during the COVID-19 pandemic in Ehlanzeni District, Mpumalanga, South Africa**

Thandiwe Elsie Mbira^1,2^, Tendesayi Kufa^2,3^, Gayle Gillian Sherman^3,4^, Nobubelo Kwanele Ngandu^1§^ on behalf of the PHANGISA study team.

1. HIV and Other Infectious Diseases Research Unit, South African Medical Research Council, Cape Town, South Africa
2. Division of Epidemiology and Biostatistics, School of Public Health, University of the Witwatersrand, Johannesburg, South Africa
3. Centre for HIV and STIs, National Institute for Communicable Diseases, Johannesburg, South Africa.
4. Department of Paediatrics and Child Health, Faculty of Health Sciences, University of the Witwatersrand, Johannesburg, South Africa.

^§^ Corresponding author: Nobubelo Kwanele Ngandu: [Nobubelo.Ngandu@mrc.ac.za](mailto:Nobubelo.Ngandu@mrc.ac.za)

E-mail addresses and ORCID of authors:

TEM: [Thandiwe.Mbira@mrc.ac.za](mailto:Thandiwe.Mbira@mrc.ac.za) (ORCID: 0000-0001-6992-7902)

TK: [TendesayiKC@nicd.ac.za](mailto:TendesayiKC@nicd.ac.za) (ORCID: 0000-0002-9797-616X)

GGS: [Gayles@nicd.ac.za](mailto:Gayles@nicd.ac.za) (ORCID: 0000-0003-3208-5216)

NKN: [Nobubelo.Ngandu@mrc.ac.za](mailto:Nobubelo.Ngandu@mrc.ac.za) (ORCID: 0000-0001-8883-3821)

**Table S1A:** Distribution of compliance to repeat VL tests during pre-COVID-19 (N=282), transition (N=211), and COVID-19 (N=207) periods using 2015 or 2019 guidelines by demographic and socioeconomic factors

|  | Pre-COVID-19 | | | | Transitional Period | | | | COVID-19 | | | |
| --- | --- | --- | --- | --- | --- | --- | --- | --- | --- | --- | --- | --- |
|  |  | On-time | Delayed/  Missed | Chi-sq p value |  | On-time | Delayed/  Missed | Chi-sq p value |  | On-time | Delayed/  Missed | Chi-sq p value |
|  | N | %  [95% CI] | %  [95% CI] |  | N | %  [95% CI] | %  [95% CI] |  | N | %  [95% CI] | %  [95% CI] |  |
| All | 282 | 82.6  [77.8, 86.6] | 17.4  [13.4, 22.2] |  | 211 | 81.5  [75.7, 86.2] | 18.5  [13.8, 24.3] |  | 207 | 86.0  [80.5, 90.1] | 14.0  [9.9, 19.5] |  |
| Maternal Age groups |  |  |  | **0.012** |  |  |  | 0.523 |  |  |  | 0.181 |
| 15-24 years | 44 | 68.2  [53.1, 80.2] | 31.8  [19.8, 46.9] |  | 32 | 78.1  [60.6,89.2] | 21.9  [10.8, 39.4] |  | 33 | 75.8  [58.4, 87.4] | 24.2  [12.6, 41.6] |  |
| 25-34 years | 163 | 87.1  [81.0, 91.5] | 12.9  [8.5, 19.0] |  | 120 | 84.2  [76.5, 89.7] | 15.8  [10.3, 23.5] |  | 123 | 87.8  [80.7, 92.5] | 12.2  [7.5, 19.3] |  |
| 35-46 years | 75 | 81.3  [70.9, 88.6] | 18.7  [11.4, 29.1] |  | 59 | 78.0  [65.6, 86.8] | 22.0  [13.2, 34.4] |  | 51 | 88.2  [76.1, 94.6] | 11.8  [5.4, 23.9] |  |
| BMI groups |  |  |  | 0.636 |  |  |  | 0.362 |  |  |  | **0.022** |
| Obese | 99 | 82.8  [74.2, 90.5] | 17.2  [10.9, 25.9] |  | 74 | 87.8  [78.2, 93.6] | 12.2  [6.4, 21.8] |  | 74 | 86.5  [76.6, 92.6] | 13.5  [7.4, 23.4] |  |
| Overweight | 81 | 84.0  [74.2, 90.5] | 16.0  [9.5, 25.8] |  | 61 | 78.7  [66.6, 93.6] | 21.3  [12.8, 33.4] |  | 55 | 80.0  [67.3, 88.6] | 20.0  [11.4, 32.7] |  |
| Normal | 88 | 79.5  [69.8, 86.7] | 20.5  [13.3, 30.2] |  | 64 | 76.9  [65.1, 85.6] | 23.1  [14.4, 34.9] |  | 66 | 93.9  [84.9, 97.7] | 6.1  [2.3, 15.1] |  |
| Underweight | 14 | 92.9  [62.8, 99.0] | 7.1  [1.0, 37.2] |  | 11 | 81.8  [49.1, 95.5] | 18.2  [4.5, 50.9] |  | 11 | 63.6  [33.7, 85.8] | 36.4  [14.2, 66.3] |  |
| Level of Education |  |  |  | 0.198 |  |  |  | 0.303 |  |  |  | 0.962 |
| None-Grade 7 | 19 | 68.4  [45.1, 85.1] | 31.6  [14.9, 54.9] |  | 13 | 84.6  [54.7, 96.2] | 15.4  [3.8, 45.3] |  | 12 | 83.3  [52.1, 95.8] | 16.7  [4.2, 47.9] |  |
| Grade 8-12 | 160 | 82.5  [75.8, 87.7] | 17.5  [12.3, 24.2] |  | 123 | 78.0  [69.8, 84.5] | 22.0  [15.5, 30.2] |  | 122 | 86.1  [78.7, 91.2] | 13.9  [8.8, 21.3] |  |
| Over high school | 103 | 85.4  [77.2, 91.0] | 14.6  [9.0, 22.8] |  | 75 | 86.7  [76.9, 92.7] | 13.3  [7.3, 23.1] |  | 73 | 86.3  [76.3, 92.5] | 13.7  [7.5, 23.7] |  |
| Marital status |  |  |  | 0.932 |  |  |  | 0.478 |  |  |  | 0.649 |
| Never married/ not cohabiting | 161 | 82.6  [75.9, 87.7] | 17.4  [12.3, 24.1] |  | 132 | 79.5  [71.8, 85.6] | 20.5  [14.4, 28.2] |  | 115 | 84.3  [76.5, 89.9] | 15.7  [10.1, 23.5] |  |
| Married/cohabiting | 113 | 82.3  [74.1, 88.3] | 17.7  [11.7, 25.9] |  | 71 | 85.9  [75.7, 92.3] | 14.1  [7.7, 24.3] |  | 87 | 88.5  [79.9, 93.7] | 11.5  [6.3, 20.1] |  |
| Widowed/divorced/separate | 8 | 87.5  [46.0, 98.3] | 12.5  [1.7, 54.0] |  | 8 | 75.0  [37.5, 93.8] | 25.0  [6.2, 62.5] |  | 5 | 80.0  [30.6, 97.3] | 20.0  [2.7, 69.4] |  |
| Source of income |  |  |  | 0.992 |  |  |  | 0.871 |  |  |  | 0.954 |
| Employed | 82 | 81.7  [71.8, 88.7] | 18.3  [11.3, 28.2] |  | 52 | 80.8  [67.7, 89.4] | 19.2  [10.6, 32.3] |  | 59 | 84.7 [73.1, 91.9] | 15.3 [8.1, 26.9] |  |
| Spouse/partner | 81 | 82.7  [72.9, 89.5] | 17.3  [10.5, 27.1] |  | 58 | 79.3  [66.9, 87.9] | 20.7  [12.1, 33.1] |  | 63 | 87.3 [76.5, 93.5] | 12.7 [6.5, 33.5] |  |
| Depend on others | 40 | 82.5  [67.5, 91.4] | 17.5  [8.6, 32.5] |  | 36 | 86.1  [70.6, 94.1] | 13.9  [5.9, 29.4] |  | 31 | 83.9 [66.5, 93.2] | 16.1 [6.8, 33.5] |  |
| Government grant/no income | 79 | 83.5  [73.6, 90.2] | 16.5  [9.8, 26.4] |  | 65 | 81.5  [70.2, 89.2] | 18.5  [10.8, 29.8] |  | 54 | 87.0 [75.1, 93.7] | 13.0 [6.3, 24.9] |  |
| Monthly income |  |  |  | 0.169 |  |  |  | 0.725 |  |  |  | 0.970 |
| No income- R3200 | 163 | 85.3  [78.9, 89.9] | 14.7  [10.1, 21.1] |  | 135 | 82.2  [74.8, 87.8] | 17.8  [12.2, 25.2] |  | 85 | 85.9  [76.7, 91.8] | 14.1  [8.2, 23.3] |  |
| >R3200 | 119 | 79.0  [70.7, 85.4] | 21.0  [14.6, 29.3] |  | 76 | 80.3  [69.7, 87.8] | 19.7  [12.2, 30.3] |  | 122 | 86.1  [78.7, 91.2] | 13.9  [8.8, 21.3] |  |
| Partner's HIV status |  |  |  | 0.503 |  |  |  | 0.240 |  |  |  | 0.672 |
| Positive | 141 | 80.1  [72.7, 85.9] | 19.9  [14.1, 27.3] |  | 97 | 77.3  [67.9, 84.6] | 22.7  [15.4, 32.1] |  | 101 | 88.1  [80.2, 93.2] | 11.9  [6.8, 19.8] |  |
| Negative | 46 | 87.0  [73.8, 94.0] | 13.0  [6.0, 26.2] |  | 42 | 81.0  [66.2, 90.2] | 19.0  [9.8, 33.8] |  | 35 | 82.9  [66.6, 92.1] | 17.1  [7.9, 33.4] |  |
| Unknown | 95 | 84.2  [75.4, 90.3] | 15.8  [9.7, 24.6] |  | 72 | 87.5  [77.6, 93.4] | 12.5  [6.6, 22.4] |  | 71 | 84.5  [74.1, 91.2] | 15.5  [8.8, 25.9] |  |
| Condom use frequency* |  |  |  | 0.998 |  |  |  | 0.900 |  |  |  | 0.984 |
| Never | 23 | 82.6  [61.7, 93.3] | 17.4  [6.7, 38.3] |  | 20 | 80.0  [57.1, 92.3] | 20.0  [7.7, 42.9] |  | 15 | 86.7  [59.2, 96.7] | 13.3  [3.3, 40.8] |  |
| Sometimes | 110 | 82.7  [74.5, 88.7] | 17.3  [11.3; 25.5] |  | 88 | 83.0  [73.6, 89.5] | 17.0  [10.5, 26.4] |  | 81 | 86.4  [77.1, 92.3] | 13.5  [7.7, 22.9] |  |
| Always | 147 | 83.0  [76.0, 88.3] | 17.0  [11.7, 24.0] |  | 103 | 80.6  [71.7, 87.1] | 19.4  [12.9, 28.3] |  | 111 | 85.6  [77.7, 91.0] | 14.4  [9.0, 22.3] |  |
| Planned pregnancy |  |  |  | 0.542 |  |  |  | 0.812 |  |  |  | 0.333 |
| No | 150 | 81.3  [74.3, 86.8] | 18.7  [13.2, 25.7] |  | 110 | 80.9  [72.4, 87.2] | 19.1  [12.8, 27.6] |  | 110 | 88.2  [80.7, 93.0] | 11.8  [7.0, 19.3] |  |
| Yes | 132 | 84.1  [76.8, 89.4] | 15.9  [10.6, 23.2] |  | 101 | 82.2  [73.4, 88.5] | 17.8  [11.5, 26.6] |  | 97 | 83.5  [74.7, 89.7] | 16.5  [10.3, 25.3] |  |
| Lockdown levels at a repeat test |  |  |  |  |  |  |  |  |  |  |  | 0.832 |
| Level 1 |  |  |  |  |  |  |  |  | 42 | 88.1  [74.3, 95.0] | 11.9  [5.0, 25.7] |  |
| Level 2 |  |  |  |  |  |  |  |  | 22 | 81.8  [60.2, 93.0] | 18.2  [7.0, 39.8] |  |
| Level 3 |  |  |  |  |  |  |  |  | 72 | 88.9  [79.3, 94.4] | 11.1  [5.6, 20.7] |  |
| Level 4 |  |  |  |  |  |  |  |  | 30 | 83.3  [65.6, 92.9] | 16.7  [7.1, 34.4] |  |
| Level 5 |  |  |  |  |  |  |  |  | 41 | 82.9  [68.2, 91.7] | 17.1  [8.3, 31.8] |  |

* Variables with missing data, Bolded p values= statistically significant. n=number of cases per category; BMI, Body mass index; ART, Antiretroviral treatment; ANC, Antenatal Care; PVT, Prevention of vertical HIV transmission

**Table S1B:** Distribution of compliance to repeat VL tests during pre-COVID-19 (N=282), transition (N=211), and COVID-19 (N=207) periods using 2015 or 2019 guidelines by PVT factors

|  | Pre-COVID-19 | | | | | Transitional Period | | | | COVID-19 | | | |
| --- | --- | --- | --- | --- | --- | --- | --- | --- | --- | --- | --- | --- | --- |
|  |  | On-time | | Delayed/ Missed | Chi-sq  p value |  | On-time | Delayed/ Missed | Chi-sq p value |  | On-time | Delayed/Missed | Chi-sq p value |
|  | N | %  [95% CI] | | %  [95% CI] |  | N | %  [95% CI] | %  [95% CI] |  | N | %  [95% CI] | %  [95% CI] |  |
| PVT stages at baseline |  |  | |  | 0.161 |  |  |  | 0.656 |  |  |  | 0.389 |
| Pregnant | 87 | 87.4  [78.5, 92.9] | | 12.6  [7.1, 21.5] |  | 75 | 80.0  [69.4, 87.6] | 20.0  [12.4, 30.6] |  | 51 | 82.4  [69.3, 90.6] | 17.6  [9.4, 30.7] |  |
| Postpartum | 195 | 80.5  [74.3, 85.5] | | 19.5  [14.5, 25.7] |  | 136 | 82.4  [75.0, 87.9] | 17.6  [12.1, 25.0] |  | 156 | 87.2  [80.9, 91.6] | 12.8  [8.4, 19.1] |  |
| Previous VL |  |  | |  | **<0.001** |  |  |  | **<0.001** |  |  |  | **<0.001** |
| VL<50 copies/ml | 198 | | 91.4  [86.6, 94.6] | 8.6  [5.4, 13.4] |  | 186 | 86.6  [80.8, 90.8] | 13.4  [9.2, 19.2] |  | 172 | 97.7  [93.9, 99.1] | 2.3  [0.9, 6.1] |  |
| VL=50-999 copies/ml | 60 | | 81.7  [69.8, 89.6] | 18.3  [10.4, 30.2] |  | 21 | 42.9  [23.9, 64.2] | 57.1  [35.8, 76.1] |  | 21 | 38.1  [20.2, 59.9] | 61.9  [40.1,79.7] |  |
| VL≥1000 copies/ml | 24 | | 12.5  [4.1, 32.5] | 87.5  [67.5, 95.9] |  | 4 | 50.0  [12.2, 87.8] | 50.0  [12.2, 87.8] |  | 14 | 14.3  [3.6, 42.9] | 85.7  [57.1,96.4] |  |
| Gestation at first ANC visit |  |  | |  | 0.619 |  |  |  | 0.854 |  |  |  | 0.860 |
| 0-12 weeks | 178 | 84.3  [78.1, 88.9] | | 15.7  [11.1, 21.9] |  | 124 | 83.1  [75.4, 88.7] | 16.9  [11.3,24.6] |  | 137 | 85.4  [78.4, 90.4] | 14.6  [9.6, 21.6] |  |
| 13-20 weeks | 81 | 80.2  [70.1, 87.6] | | 19.8  [12.4, 29.9] |  | 69 | 79.7  [68.5, 87.6] | 20.3  [12.4, 31.5] |  | 51 | 88.2  [76.1, 94.6] | 11.8  [5.4, 23.9] |  |
| 21-40 weeks | 23 | 78.3  [57.1, 90.7] | | 21.7  [9.3, 42.9] |  | 18 | 77.8  [53.4, 91.5] | 22.2  [8.5, 46.6] |  | 19 | 84.2  [60.7, 94.9] | 15.8  [5.1, 39.3] |  |
| Number of ANC visits |  |  | |  | 0.181 |  |  |  | 0.995 |  |  |  | 0.658 |
| 0-4 visits | 103 | 78.6  [69.6, 85.5] | | 21.4  [14.5, 30.4] |  | 76 | 81.6  [71.2, 88.8] | 18.4  [11.2, 28.8] |  | 78 | 84.6  [74.8, 91.1] | 15.4  [8.9, 25.2] |  |
| 5-12 visits | 179 | 84.9  [78.9, 89.5] | | 15.1  [10.5, 21.1] |  | 135 | 81.5  [74.0, 87.2] | 18.5  [12.8, 26.0] |  | 129 | 86.8  [79.8, 91.7] | 13.2  [8.3, 20.2] |  |
| HIV diagnosis |  |  | |  | 0.418 |  |  |  | 0.504 |  |  |  | 0.244 |
| Before pregnancy | 225 | 81.3  [75.7, 85.9] | | 18.7  [14.1, 24.3] |  | 160 | 80.0  [73.0, 85.5] | 20.0  [14.5, 27.0] |  | 146 | 88.4  [82.0, 92.7] | 11.6  [7.3, 18.0] |  |
| During pregnancy, before 28 weeks | 53 | 86.8  [74.7, 93.6] | | 13.2  [6.4, 25.3] |  | 46 | 84.8  [71.3, 92.6] | 15.2  [7.4, 28.7] |  | 54 | 81.5  [68.8, 89.8] | 18.5  [10.2, 31.2] |  |
| During pregnancy, after 28 weeks or during delivery or postnatal | 4 | 100 | | 0 |  | 5 | 100 | 0 |  | 7 | 71.4  [32.4, 92.9] | 28.6  [7.1, 67.6] |  |
| ART initiation |  |  | |  | 0.670 |  |  |  | 0.476 |  |  |  | 0.338 |
| Before pregnancy | 223 | 81.6  [76.0, 86.2] | | 18.4  [13.8, 24.0] |  | 160 | 80.0  [73.0, 85.5] | 20.0  [14.5, 27.0] |  | 145 | 88.3  [81.9, 92.6] | 11.7  [7.4, 18.1] |  |
| During pregnancy, before 28 weeks | 50 | 86.0  [73.4, 93.2] | | 14.0  [6.8, 26.6] |  | 44 | 84.1  [70.1, 92.3] | 15.9  [7.7, 29.9] |  | 53 | 81.1  [68.3, 89.6] | 18.9  [10.4, 31.7] |  |
| During pregnancy, after 28 weeks or during delivery or postnatal | 9 | 88.9  [49.8, 98.5] | | 11.1  [1.5, 50.2] |  | 7 | 100 | 0 |  | 9 | 77.8  [41.9, 94.4] | 22.2  [5.6, 58.1] |  |
| Current ART regimen* |  |  | |  | 0.438 |  |  |  | **0.014** |  |  |  | 0.784 |
| First line | 227 | 83.7  [78.3, 88.0] | | 16.3  [12.0, 21.7] |  | 177 | 78.5  [71.8, 84.0] | 21.5  [16.0, 28.2] |  | 174 | 86.2  [80.2, 90.6] | 13.8  [9.4, 19.8] |  |
| 2nd/3rd line/unknown | 53 | 79.2  [66.2, 88.1] | | 20.8  [11.9, 33.8] |  | 33 | 97.0  [81.2, 99.6] | 3.0  [0.4, 18.8] |  | 32 | 84.4  [67.4, 93.3] | 15.6  [6.6, 32.6] |  |
| Missed an ART dose last 7days |  |  | |  | 0.476 |  |  |  | 0.736 |  |  |  | 0.576 |
| No | 270 | 83.0  [78.0, 87.0] | | 17.0  [13.0, 22.0] |  | 203 | 81.8  [75.8, 86.5] | 18.2  [13.5, 24.2] |  | 197 | 86.3  [80.7, 90.5] | 13.7  [9.5, 19.3] |  |
| Yes | 12 | 75.0  [44.7, 91.8] | | 25.0  [8.2, 55.3] |  | 8 | 75.0  [37.5, 93.8] | 25.0  [6.2, 62.5] |  | 10 | 80.0  [45.7, 95.0] | 20.0  [5.0, 54.3] |  |
| Facing any ART adherence challenges |  |  | |  | 0.504 |  |  |  | 0.545 |  |  |  | 0.935 |
| No | 81 | 80.2  [70.1, 87.6] | | 19.8  [12.4, 29.9] |  | 70 | 82.9  [72.1, 90.0] | 17.1  [10.0, 27.9] |  | 70 | 85.7  [75.4, 92.2] | 14.3  [7.8, 24.6] |  |
| Yes | 201 | 83.6  [77.8, 88.1] | | 16.4  [11.9, 22.2] |  | 141 | 80.9  [73.5, 86.6] | 19.1  [13.4, 26.5] |  | 137 | 86.1  [79.2, 91.0] | 13.9  [9.0, 20.8] |  |

* Variables with missing data, Bolded p values= statistically significant. n=number of cases per category; ART, Antiretroviral treatment; ANC, Antenatal Care; PVT, Prevention of vertical HIV transmission

**Table S2A:** Distribution of compliance to repeat VL tests during pre-COVID-19 (N=280), transition (N=208), and COVID-19 (N=207) periods using 2015 guidelines by demographic and socioeconomic factors

|  | Pre-COVID-19 | | | | Transitional Period | | | | COVID-19 | | | |
| --- | --- | --- | --- | --- | --- | --- | --- | --- | --- | --- | --- | --- |
|  |  | On-time | Delayed/  Missed | Chi-sq p value |  | On-time | Delayed/  Missed | Chi-sq p value |  | On-time | Delayed/  Missed | Chi-sq p value |
|  | N | %  [95% CI] | %  [95% CI] |  | N | %  [95% CI] | %  [95% CI] |  | N | %  [95% CI] | %  [95% CI] |  |
| All | 280 | 82.9  [78.0, 86.8] | 17.1  [13.2, 22.0] |  | 208 | 86.5  [81.2, 90.6] | 13.5  [9.4, 18.8] |  | 207 | 92.3  [87.7, 95.2] | 7.7  [4.8, 12.3] |  |
| Maternal Age groups |  |  |  | **0.007** |  |  |  | 0.364 |  |  |  | 0.562 |
| 15-24 years | 43 | 67.4  [52.2, 79.7] | 32.6  [20.3, 47.8] |  | 33 | 78.8  [61.6, 89.6] | 21.2  [10.4, 38.4] |  | 33 | 87.9  [71.7, 95.4] | 12.1  [4.6, 28.3] |  |
| 25-34 years | 162 | 87.7  [81.6, 91.9] | 12.4  [8.1, 18.4] |  | 117 | 88.0  [80.8, 92.8] | 12.0  [7.2, 19.2] |  | 123 | 93.5  [87.5, 96.7] | 6.5  [3.3, 12.5] |  |
| 35-46 years | 75 | 81.3  [70.9, 88.6] | 18.7  [11.4, 29.1] |  | 58 | 87.9  [76.7, 94.2] | 12.1  [5.8, 23.3] |  | 51 | 92.2  [80.8, 97.0] | 7.8  [3.0, 19.2] |  |
| BMI groups |  |  |  | 0.625 |  |  |  | 0.705 |  |  |  | 0.126 |
| Obese | 98 | 83.7  [75.0, 89.8] | 16.3  [10.2, 25.0] |  | 73 | 89.0  [79.5, 94.4] | 11.0  [5.6, 20.5] |  | 74 | 93.2  [84.7, 97.2] | 6.8  [2.8, 15.3] |  |
| Overweight | 80 | 83.8  [73.9, 90.3] | 16.3  [9.7, 26.1] |  | 59 | 88.1  [77.1, 94.3] | 11.9  [5.7, 22.9] |  | 55 | 87.3  [75.5, 93.8] | 12.7  [6.2, 24.5] |  |
| Normal | 88 | 79.6  [69.8, 86.7] | 20.5  [13.3, 30.2] |  | 65 | 83.1  [71.9, 90.4] | 16.9  [9.6, 28.1] |  | 66 | 97.0  [88.6, 99.2] | 3.0  [0.75, 11.4] |  |
| Underweight | 14 | 92.9  [62.8, 99.0] | 7.1  0.10, 37.2] |  | 11 | 81.8  [49.1, 95.5] | 18.2  [4.5, 50.9] |  | 11 | 81.8  [49.1, 95.5] | 18.2  [4.5, 50.9] |  |
| Level of Education |  |  |  | 0.195 |  |  |  | 0.768 |  |  |  | 0.486 |
| None-Grade 7 | 19 | 68.4  [45.1, 85.1] | 31.6  [14.9, 54.9] |  | 13 | 84.6  [54.7, 96.2] | 15.4  [3.8, 45.3] |  | 12 | 83.3  [52.1, 95.8] | 16.7  [4.2, 47.9] |  |
| Grade 8-12 | 158 | 82.9  [76.2, 88.0] | 17.1  [12.0, 23.8] |  | 123 | 85.4  [77.9, 90.6] | 14.6  [9.4, 22.1] |  | 122 | 92.6  [86.4, 96.1] | 7.4  [3.9, 13.6] |  |
| Over high school | 103 | 85.4  [77.2, 91.0] | 14.6  [9.0, 22.8] |  | 72 | 88.9  [79.3, 94.4] | 11.1  [5.6, 20.7] |  | 73 | 93.2  [84.5, 97.1] | 6.9  [2.9, 15.5] |  |
| Marital status |  |  |  | 0.928 |  |  |  | 0.269 |  |  |  | 0.559 |
| Never married/ not cohabiting | 159 | 83.0  [76.3, 88.1] | 17.0  [11.9, 23.7] |  | 131 | 84.7  [77.5, 90.0] | 15.3  [10.0, 22.5] |  | 115 | 93.0  [86.7, 96.5] | 7.0  [3.5, 13.3] |  |
| Married/cohabiting | 113 | 82.3  [74.1, 88.3] | 17.7  [11.7, 25.9] |  | 69 | 91.3  [81.9, 96.1] | 8.7  [3.9, 18.1] |  | 87 | 92.0  [84.0, 96.1] | 8.0  [3.9, 16.0] |  |
| Widowed/divorced/separate | 8 | 87.5  [46.0, 98.3] | 12.5  [1.7, 54.0] |  | 8 | 75.0  [37.5, 93.8] | 25.0  [6.2, 62.5] |  | 5 | 80.0  [30.6, 97.3 | 20.0  [2.7, 69.4] |  |
| Source of income |  |  |  | 0.983 |  |  |  | 0.653 |  |  |  | 0.701 |
| Employed | 82 | 81.7  [71.8, 88.7] | 18.3  [11.3, 28.2] |  | 51 | 90.2  [78.4, 95.9] | 9.8  [4.1, 21.6] |  | 59 | 93.2  [83.2, 97.4] | 6.8  [2.5, 16.8] |  |
| Spouse/partner | 80 | 83.8  [73.9, 90.3] | 16.3  [9.7, 26.1] |  | 58 | 86.2  [74.7, 93.0] | 13.8  [7.0, 25.3] |  | 63 | 90.5  [80.3, 95.7] | 9.5  [4.3, 19.7] |  |
| Depend on others | 39 | 82.1  [66.8, 91.2] | 18.0  [8.8, 33.2] |  | 36 | 88.9  [73.8, 95.8] | 11.1  [4.2, 26.2] |  | 31 | 96.8  [80.3, 95.7] | 3.2  [0.4, 19.8] |  |
| Government grant/no income | 79 | 83.5  [73.6, 90.2] | 16.5  [9.8, 26.4] |  | 63 | 82.5  [71.1, 90.1] | 17.5  [9.9, 28.9] |  | 54 | 90.7  [79.5, 96.1] | 9.3  [3.9, 20.5] |  |
| Monthly income |  |  |  | 0.140 |  |  |  | 0.702 |  |  |  | 0.763 |
| No income- R3200 | 119 | 79.0  [70.7, 85.4] | 21.0  [14.9, 29.3] |  | 75 | 85.3  [75.4, 91.7] | 14.7  [8.3, 24.6] |  | 85 | 92.9  [85.1, 96.8] | 7.1  [3.2, 14.9] |  |
| >R3200 | 161 | 85.7  [79.4, 90.3] | 14.3  [9.7, 20.6] |  | 133 | 87.2  [80.4, 91.9] | 12.8  [8.1, 19.6] |  | 122 | 91.8  [85.4, 95.5] | 8.2  [4.5, 14.6] |  |
| Partner's HIV status |  |  |  | 0.580 |  |  |  | 0.283 |  |  |  | 0.132 |
| Positive | 140 | 80.7  [73.3, 86.5] | 19.3  [13.5, 26.7] |  | 95 | 84.2  [75.4, 90.3] | 15.8  [9.7, 24.6] |  | 101 | 94.1  [87.3, 97.3] | 5.9  [2.7, 12.7] |  |
| Negative | 45 | 87.0  [73.8, 94.0] | 13.0  [6.0, 26.2] |  | 41 | 82.9  [68.2, 91.7] | 17.1  [8.3, 31.8] |  | 35 | 97.1  [82.1, 99.6] | 2.9  [0.4, 17.9] |  |
| Unknown | 94 | 84.0  [75.2, 90.2] | 16.0  [9.8, 24.8] |  | 72 | 91.7  [82.6, 96.2] | 8.3  [3.8, 17.4] |  | 71 | 87.3  [77.3, 93.3] | 12.7  [6.7, 22.7] |  |
| Condom use frequency* |  |  |  | 0.990 |  |  |  | 0.596 |  |  |  | 0.164 |
| Never | 23 | 82.6  [61.7, 93.3] | 17.4  [6.7, 38.3] |  | 19 | 78,9  [55.3, 91.9] | 21.1  [8.1, 44.7] |  | 15 | 100.0 | 0.0 |  |
| Sometimes | 109 | 83.5  [75.3, 89.4] | 16.5  [10.6, 24.7] |  | 87 | 87.4  [78.5, 92.9] | 12.6  [7.1, 21.5] |  | 81 | 95.1  [87.5, 98.1] | 4.9  [1.9, 12.5] |  |
| Always | 146 | 82.9  [75.8, 88.2] | 17.1  [11.8, 24.2] |  | 102 | 87.3  [79.2, 92.5] | 12.8  [7.5, 20.8] |  | 111 | 89.2  [81.9, 93.8] | 10.8  [6.2, 18.1] |  |
| Planned pregnancy |  |  |  | 0.435 |  |  |  | 0.938 |  |  |  | 0.192 |
| No | 149 | 81.2  [74.1, 86.7] | 18.8  [13.3, 25.9] |  | 110 | 86.4  [78.6, 91.6] | 13.6  [8.4, 21.1] |  | 110 | 94.6  [88.3, 97.5] | 5.5  [2.5, 11.7] |  |
| Yes | 131 | 84.7  [77.5, 90.0] | 15.3  [10.0, 22.5] |  | 98 | 86.7  [78.4, 92.2] | 13.3  [7.8, 21.6] |  | 97 | 89.7  [81.8, 94.4] | 10.3  [5.6, 18.2] |  |
| Lockdown levels at a repeat test |  |  |  |  |  |  |  | 0.938 |  |  |  | 0.790 |
| Level 1 |  |  |  |  |  |  |  |  | 94 | 94.7  [87.8, 97.8] | 5.3  [2.2, 12.2] |  |
| Level 2 |  |  |  |  |  |  |  |  | 25 | 92.0  [72.9, 98.0] | 8.0  [2.0, 27.1] |  |
| Level 3 |  |  |  |  |  |  |  |  | 69 | 89.9  [80.1, 95.1] | 10.1  [4.9, 19.9] |  |
| Level 4 |  |  |  |  |  |  |  |  | 18 | 88.9  [64.6, 97.2] | 11.1  [2.8, 35.4] |  |
| Level 5 |  |  |  |  |  |  |  |  | 1 | 100.0 | 0.0 |  |

* Variables with missing data, Bolded p values= statistically significant. n=number of cases per category; BMI, Body mass index; ART, Antiretroviral treatment; ANC, Antenatal Care; PVT, Prevention of vertical HIV transmission

**Table S2B:** Distribution of compliance to repeat VL tests during pre-COVID-19 (N=282), transition (N=211), and COVID-19 (N=207) periods using 2015 guidelines by PVT factors

|  | Pre-COVID-19 | | | | Transitional Period | | | | COVID-19 | | | | | |
| --- | --- | --- | --- | --- | --- | --- | --- | --- | --- | --- | --- | --- | --- | --- |
|  |  | On-time | Delayed/ Missed | Chi-sq  p value |  | On-time | Delayed/ Missed | Chi-sq p value |  | On-time | Delayed/Missed | | Chi-sq p value | |
|  |  |  |  |  |  |  |  |  |  |  |  | |  | |
|  | N | %  [95% CI] | %  [95% CI] |  | N | %  [95% CI] | %  [95% CI] |  | N | %  [95% CI] | %  [95% CI] | |  | |
| PVT stages at baseline |  |  |  | 0.198 |  |  |  | 0.941 |  |  |  | | 0.523 | |
| Pregnant | 86 | 87.2  [78.3, 92.8] | 12.8  [7.2, 21.7] |  | 73 | 86.3  [76.3, 92.5] | 13.7  [7.5, 23.7] |  | 51 | 90.2  [78.4, 95.9] | 9.8  [4.1, 21.6] | |  | |
| Postpartum | 194 | 80.9  [74.6, 85.9] | 19.1  [14.1, 25.2] |  | 135 | 86.7  [79.8, 91.5] | 13.3  [8.5, 20.2] |  | 156 | 92.9  [87.7, 96.1] | 7.1  [3.9, 12.3] | |  | |
| Previous VL |  |  |  | **<0.001** |  |  |  | **0.003** |  |  |  | | **<0.001** | |
| VL<50 copies/ml | 198 | 91.4  [86.6, 94.6] | 8.6  [5.4, 13.4] |  | 153 | 83.7  [76.9, 88.7] | 16.3  [11.3, 23.1] |  | 172 | 97.7  [93.9, 99.1] | 2.3  [0.9, 6.1] | | |  |
| VL=50-999 copies/ml | 58 | 82.8  [70.8, 90.5 | 17.2  [9.5, 29.2] |  | 54 | 96.3  [86.3, 99.1] | 3.7  [0.9, 13.7] |  | 21 | 100.0 | 0.0 |  | | |
| VL≥1000 copies/ml | 24 | 12.5  [4.1, 32.5] | 87.5  [67.5, 95.9] |  | 1 | 0.0 | 100.0 |  | 14 | 14.3  [3.6, 43.0] | 85.7  [57.1, 96.4] | | |  |
| Gestation at first ANC visit |  |  |  | 0.568 |  |  |  | 0.514 |  |  |  | | | 0.885 |
| 0-12 weeks | 176 | 84.7  [78.5, 89.3] | 15.3  [10.7, 21.5] |  | 122 | 87.7  [80.6, 92.5] | 12.3  [7.5, 19.4] |  | 137 | 92.7  [86.9, 96.0] | 7.3  [4.0, 13.1] | |  | |
| 13-20 weeks | 81 | 80.3  [70.1, 87.6] | 19.8  [12.4, 29.9] |  | 68 | 86.8  [76.4, 93.0] | 13.2  [7.0, 23.6] |  | 51 | 92.2  [80.8, 97.0] | 7.8  [3.0, 19.2] | |  | |
| 21-40 weeks | 23 | 78.3  [57.1, 90.7] | 21.7  [9.3, 42.9] |  | 18 | 77.8  [53.4, 91.5] | 22.2  [8.5, 92.5] |  | 19 | 89.5  [66.1, 97.4] | 10.5  [2.6, 33.9] | |  | |
| Number of ANC visits |  |  |  | 0.137 |  |  |  | 0.142 |  |  |  | | 0.581 | |
| 0-4 visits | 102 | 78.4  [69.4, 85.] | 21.6  [14.6, 30.6] |  | 78 | 91.0  [82.3, 95.7] | 9.0  [4.3, 17.7] |  | 78 | 93.6  [85.4, 97.3] | 6.4  [2.7, 14.6] | |  | |
| 5-12 visits | 178 | 85.4  [79.4, 89.9] | 14.6  [10.1, 20.6] |  | 130 | 83.9  [76.4, 89.3] | 16.2  [10.7, 23.6] |  | 129 | 91.5  [85.2, 95.2] | 8.5  [4.8, 14.8] | |  | |
| HIV diagnosis |  |  |  | 0.464 |  |  |  | 0.660 |  |  |  | | 0.447 | |
| Before pregnancy | 224 | 81.7  [76.1, 86.2] | 18.3  [13.8, 23.9] |  | 155 | 86.5  [80.1, 91.0] | 13.6  [9.0, 19.9] |  | 146 | 93.2  [87.7, 96.3] | 6.8  [3.7, 12.3] | |  | |
| During pregnancy, before 28 weeks | 52 | 86.5  [74.3, 93.5] | 13.5  [6.5, 25.7] |  | 48 | 85.4  [72.3, 92.9] | 14.6  [7.1, 27.7] |  | 54 | 88.9  [77.3, 94.9] | 11.1  [5.1, 22.7] | |  | |
| During pregnancy, after 28 weeks or during delivery or postnatal | 4 | 100.0 | 0.0 |  | 5 | 100.0 | 0.0 |  | 7 | 100.0 | 0.0 | |  | |
| ART initiation |  |  |  | 0.729 |  |  |  | 0.546 |  |  |  | | 0.396 | |
| Before pregnancy | 222 | 82.0  [76.3, 86.5] | 18.0  [13.7, 23.9] |  | 155 | 86.5  [80.1, 91.0] | 13.5  [9.0, 19.9] |  | 145 | 93.1  [87.6, 96.3] | 6.9  [3.7, 12.4] | |  | |
| During pregnancy, before 28 weeks | 49 | 85.7  [72.9, 93.1] | 14.3  [6.9, 27.1] |  | 46 | 84.8  [71.3, 92.6] | 15.2  [7.4, 28.7] |  | 53 | 88.7  [76.9, 94.8] | 11.3  [5.2, 23.1] | |  | |
| During pregnancy, after 28 weeks or during delivery or postnatal | 9 | 88.9  [49.8, 98.5] | 11.1  [1.5, 50.2] |  | 7 | 100.0 | 0.0 |  | 9 | 100.0 | 0.0 | |  | |
| Current ART regimen* |  |  |  | 0.406 |  |  |  | 0.069 |  |  |  | | 0.286 | |
| First line | 225 | 84.0  [78.6, 88.2] | 16.0  [11.8, 21.4] |  | 176 | 84.7  [78.5, 89.3] | 15.3  [10.7, 21.5] |  | 174 | 91.4  [86.2, 94.8] | 8.6  [5.2, 13.8] | |  | |
| 2^nd^/3^rd^ line/unknown | 53 | 79.3  [66.2, 88.1] | 20.8  [11.9, 33.8] |  | 31 | 96.8  [80.2, 99.6] | 3.2  [0.4, 19.8] |  | 32 | 96.9  [80.7, 99.6] | 3.1  [0.4, 19.3] | |  | |
| Missed an ART dose last 7days |  |  |  | 0.460 |  |  |  | 0.935 |  |  |  | | 0.783 | |
| No | 268 | 83.2  [78.2, 87.2] | 16.8  [12.8, 21.8] |  | 200 | 86.5  [81.0, 90.6] | 13.5  [9.4, 19.0] |  | 197 | 92.4  [87.7, 95.4] | 7.6  [4.6, 12.3] | |  | |
| Yes | 12 | 75.0  [44.7, 91.8] | 25.0  [8.2, 55.3] |  | 8 | 87.5  [46.0, 98.3] | 12.5  [1.7, 54.0] |  | 10 | 90.0  [53.0, 98.6] | 10.0  [1.4, 47.0] | |  | |
| Facing any ART adherence challenges |  |  |  | 0.652 |  |  |  | 0.498 |  |  |  | | 0.382 | |
| No | 80 | 81.3  [71.1, 88.4] | 18.8  [11.6, 28.9] |  | 70 | 84.3  [73.7, 91.1] | 15.7  [8.9, 26.3] |  | 70 | 90.0  [80.4, 95.2] | 10.0  [4.8, 19.6] | |  | |
| Yes | 200 | 83.5  [77.7, 88.0] | 16.5  [12.0, 22.3] |  | 138 | 87.7  [81.0, 92.2] | 12.3  [7.8, 19.0] |  | 137 | 93.4  [87.8, 96.6] | 6.6  [3.4, 12.2] | |  | |

* Variables with missing data, Bolded p values= statistically significant. n=number of cases per category; ART, Antiretroviral treatment; ANC, Antenatal Care; PVT, Prevention of vertical HIV transmission

**Table S3:** Factors associated with compliance to VL testing 2015 or 2019 guidelines among PVT clients across the three time periods (N=700 repeat tests)

|  | Number with on-time repeat VLs (row %) | Unadjusted | | Adjusted | |
| --- | --- | --- | --- | --- | --- |
|  |  | **IRR (95% CI)** | **Poisson p value** | **IRR (95% CI)** | **Poisson p value** |
| COVID-19 stages |  |  | 0.348 |  |  |
| Pre-COVID-19 | 233 (82.6) | Ref |  |  |  |
| Transition | 172 (81.5) | 0.99 [0.1, 1.07] | 0.752 |  |  |
| COVID-19 | 178 (86.0) | 1.04 [0.96, 1.12] | 0.308 |  |  |
| Maternal Age groups |  |  | 0.216 |  |  |
| 15-24 years | 80 (73.4) | Ref |  | Ref |  |
| 25-34 years | 351 (86.5) | 1.18 [1.05, 1.33] | 0.007 | 1.12 [1.01, 1.23] | **0.027** |
| 35-46 years | 152 (82.2) | 1.12 [0.98, 1.28] | 0.093 | 1.08 [0.97, 1.21] | 0.176 |
| BMI groups |  |  | 0.404 |  |  |
| Obese | 2111 (85.4) | Ref |  |  |  |
| Overweight | 160 (81.2) | 0.95 [0.87, 1.03] | 0.243 |  |  |
| Normal | 182 (83.1) | 0.97 [0.90, 1.05] | 0.494 |  |  |
| Underweight | 29 (80.6) | 0.94 [0.80, 1.12] | 0.495 |  |  |
| Level of Education |  |  | **0.097** |  |  |
| None-Grade 7 | 34 (77.3) | Ref |  | Ref |  |
| Grade 8-12 | 333 (82.2) | 1. 06 [0.90,1.26] | 0.465 | 1.00 [0.86, 1.15] | 0.958 |
| Over high school | 216 (86.1) | 1.11 [0.94, 1.32] | 0.209 | 1.03 [0.89, 1.20] | 0.681 |
| Marital status |  |  | 0.432 |  |  |
| Never married/ not cohabiting | 335 (82.1) | Ref |  |  |  |
| Married/cohabiting | 231 (85.2) | 1.04 [0.97, 1.11] | 0.275 |  |  |
| Widowed/divorced/separated | 17 (81.0) | 0.99 [0.80, 1.22] | 0.896 |  |  |
| Source of income |  |  | 0.677 |  |  |
| Employed | 159 (82.4) | Ref |  | Ref |  |
| Spouse/partner | 168 (83.2) | 1.01 [0.92, 1.10] | 0.837 | 0.98 [0.91, 1.06] | 0.623 |
| Depend on others | 90 (84.1) | 1.02 [0.92, 1.13] | 0.699 | 1.04 [0.95, 1.13] | 0.448 |
| Government grant/no income | 166 (83.8) | 1.02 [0.93, 1.11] | 0.701 | 1.01 [0.93, 1.09] | 0.831 |
| Monthly income |  |  | 0.292 |  |  |
| >R3200 | 228 (81.4) | Ref |  | Ref |  |
| No income- R3200 | 355 (84.5) | 1.04 [0.97, 1.11] | 0.292 | 1.05 [0.99, 1.12] | 0.106 |
| Partner's HIV status |  |  | 0.247 |  |  |
| Positive | 277 (81.7) | Ref |  |  |  |
| Negative | 103 (83.7) | 1.02 [0.93, 1.12] | 0.604 |  |  |
| Unknown | 203 (85.2) | 1.04 [0.97, 1.12] | 0.249 |  |  |
| Condom use frequency* |  |  | 0.919 |  |  |
| Never | 48 (82.8) | Ref |  |  |  |
| Sometimes | 234 (83.9) | 1.01 [0.89, 1.15] | 0.838 |  |  |
| Always | 300 (83.1) | 1.00 [0.88, 1.14] | 0.949 |  |  |
| Planned pregnancy |  |  | 0.975 |  |  |
| No | 308 (83.2) | Ref |  |  |  |
| Yes | 275 (83.3) | 1.00 [0.94, 1.07] | 0.975 |  |  |
| PVT stages |  |  | 0.894 |  |  |
| Pregnant | 178 (83.6) | Ref |  |  |  |
| Postpartum | 405 (83.2) | 1.00 [0.93, 1.07] | 0.894 |  |  |
| Baseline or previous VL |  |  | **<0.001** |  |  |
| VL<50 copies/ml | 510 (91.7) | Ref |  | Ref |  |
| VL=50-999 copies/ml | 66 (64.7) | 0.78 [0.61, 0.82] | 0.000 | 0.71 [0.61, 0.82] | **<0.001** |
| VL≥1000 copies/ml | 7 (16.7) | 0.16 [0.09, 0.36] | 0.000 | 0.18 [0.09, 0.36] | **<0.001** |
| Gestation at first ANC visit |  |  | 0.342 |  |  |
| 0-12 weeks | 370 (84.3) | Ref |  |  |  |
| 13-20 weeks | 165 (82.1) | 0.97 [0.90, 1.05] | 0.498 |  |  |
| 21-40 weeks | 48 (80.0) | 0.95 [0.83, 1.08] | 0.442 |  |  |
| Number of ANC visits |  |  | 0.301 |  |  |
| 0-4 visits | 209 (81.3) | Ref |  |  |  |
| 5-12 visits | 374 (84.4) | 1.04 [0.97, 1.11] | 0.301 |  |  |
| ART initiation |  |  | 0.548 |  |  |
| Before pregnancy | 438 (83.0) | Ref |  |  |  |
| During pregnancy, before 28 weeks | 123 (83.7) | 1.01 [0.93, 1.09] | 0.835 |  |  |
| During pregnancy, after 28 weeks or during delivery or postnatal | 22 (88.0) | 1.06 [0.91, 1.23] | 0.440 |  |  |
| Current ART regimen* |  |  | 0.445 |  |  |
| First line | 479 (82.9) | Ref |  |  |  |
| 2nd/3rd line/unknown | 101 (85.6) | 1.03 [0.95, 1.12] | 0.445 |  |  |
| Missed an ART dose last 7days |  |  | 0.398 |  |  |
| No | 560 (83.6) | Ref |  |  |  |
| Yes | 23 (76.7) | 0.92 [0.75, 1.12] | 0.398 |  |  |
| Facing any ART adherence challenges |  |  | 0.819 |  |  |
| No | 183 (82.8) | Ref |  |  |  |
| Yes | 400 (83.5) | 1.01 [0.94, 1.08] | 0.819 |  |  |

*Variables with missing data, Factors with overall univariate p-value<0.2 bolded, Bolded multivariate significant p-value<0.05. IRR, Incidence rate ratio; CI, Confidence Interval; n=number of cases per category; BMI, Body mass index; ART, Antiretroviral treatment; ANC, Antenatal Care; PVT, Prevention of vertical HIV transmission

**Table S4:** Factors associated with compliance to VL testing 2015 guidelines among PVT clients across the three time periods (N=695 repeat tests)

|  | Number with on-time repeat VLs (row %) | Unadjusted | | Adjusted | |
| --- | --- | --- | --- | --- | --- |
|  |  | **IRR (95% CI)** | **Poisson p value** | **IRR (95% CI)** | **Poisson p value** |
| COVID-19 stages |  |  | **0.002** |  |  |
| Pre-COVID-19 | 232 (82.9) | Ref |  | Ref |  |
| Transition | 180 (86.5) | 1.04 [0.97, 1.13] | 0.260 | 0.97 [0.91, 1.04] | 0.396 |
| COVID-19 | 191 (92.3) | 1.11 [1.04, 1.19] | 0.001 | 1.10 [1.05, 1.15] | **<0.001** |
| Maternal Age groups |  |  | **0.127** |  |  |
| 15-24 years | 84 (77.1) | Ref |  | Ref |  |
| 25-34 years | 360 (89.6) | 1.16 [1.04, 1.29] | 0.006 | 1.10 [1.00, 1.20] | **0.035** |
| 35-46 years | 159 (86.4) | 1.12 [1.00, 1,26] | 0.056 | 1.07 [0.97, 1.17] | 0.189 |
| BMI groups |  |  | 0.477 |  |  |
| Obese | 216 (88.2) | Ref |  |  |  |
| Overweight | 167 (86.1) | 0.98 [0.91, 1.05] | 0.521 |  |  |
| Normal | 188 (85.8) | 0.97 [0.91, 1.05] | 0.460 |  |  |
| Underweight | 31 (86.1) | 0.98 [0.85, 1.12] | 0.740 |  |  |
| Level of Education |  |  | **0.100** |  |  |
| None-Grade 7 | 34 (77.3) | Ref |  | Ref |  |
| Grade 8-12 | 349 (86.6) | 1.12 [0.95, 1.32] | 0.176 | 1.06 [0.92, 1.23] | 0.413 |
| Over high school | 220 (88.7) | 1.15 [0.97, 1.36] | 0.104 | 1.08 [0.93, 1.25] | 0.292 |
| Marital status |  |  | 0.974 |  |  |
| Never married/ not cohabiting | 350 (86.4) | Ref |  |  |  |
| Married/cohabiting | 236 (87.7) | 1.02 [0.96, 1.08] | 0.617 |  |  |
| Widowed/divorced/separated | 17 (81.0) | 0.94 [0.76, 1.16] | 0.544 |  |  |
| Source of income |  |  | 0.604 |  |  |
| Employed | 168 (87.5) | Ref |  | Ref |  |
| Spouse/partner | 174 (86.6) | 0.99 [0.92, 1.07] | 0.783 | 0.98 [0.92, 1.05] | 0.527 |
| Depend on others | 94 (88.7) | 1.01 [0.93, 1.11] | 0.762 | 1.01 [0.94, 1.09] | 0.701 |
| Government grant/no income | 167 (85.2) | 0.97 [0.90, 1.05] | 0.510 | 0.97 [0.91, 1.04] | 0.431 |
| Monthly income |  |  | 0.259 |  |  |
| >R3200 | 237 (85.0) | Ref |  | Ref |  |
| No income- R3200 | 366 (88.0) | 1.04 [0.97, 1.10] | 0.259 | 1.06 [1.00, 1.12] | **0.044** |
| Partner's HIV status |  |  | 0.543 |  |  |
| Positive | 288 (85.7) | Ref |  |  |  |
| Negative | 108 (88.5) | 1.03 [0.96, 1.12] | 0.414 |  |  |
| Unknown | 207 (87.3) | 1.02 [0.95, 1.09] | 0.572 |  |  |
| Condom use frequency* |  |  | 0.685 |  |  |
| Never | 49 (86.0) | Ref |  |  |  |
| Sometimes | 244 (88.1) | 1.02 [0.91, 1.15] | 0.674 |  |  |
| Always | 309 (86.1) | 1.00 [0.89, 1.12] | 0.983 |  |  |
| Planned pregnancy |  |  | 0.972 |  |  |
| No | 320 (86.7) | Ref |  |  |  |
| Yes | 283 (86.8) | 1.00 [0.94, 1.06] | 0.972 |  |  |
| PVT stages |  |  | 0.655 |  |  |
| Pregnant | 184 (87.6) | Ref |  |  |  |
| Postpartum | 419 (86.4) | 0.99 [0.93, 1.05] | 0.655 |  |  |
| Baseline or previous VL |  |  | **<0.001** |  |  |
| VL<50 copies/ml | 477 (91.2) | Ref |  | Ref |  |
| VL=50-999 copies/ml | 121 (91.0) | 1.00 [0.94, 1.06] | 0.935 | 1.02 [0.96, 1.08] | 0.571 |
| VL≥1000 copies/ml | 5 (12.8) | 0.14 [0.06, 0.33] | <0.001 | 0.14 [0.06, 0.32] | **<0.001** |
| Gestation at first ANC visit |  |  | **0.169** |  |  |
| 0-12 weeks | 383 (88.1) | Ref |  | Ref |  |
| 13-20 weeks | 171 (85.5) | 0.97 [0.91, 1.03] | 0.389 | 0.99 [0.93, 1.04] | 0.658 |
| 21-40 weeks | 49 (81.7) | 0.93 [0.82, 1.05] | 0.238 | 0.95 [0.86, 1.06] | 0.369 |
| Number of ANC visits |  |  | 0.972 |  |  |
| 0-4 visits | 224 (86.8) | Ref |  |  |  |
| 5-12 visits | 379 (86.7) | 1.00 [0.94, 1.06] | 0.972 |  |  |
| ART initiation |  |  | 0.301 |  |  |
| Before pregnancy | 451 (86.4) | Ref |  |  |  |
| During pregnancy, before 28 weeks | 128 (86.5) | 1.00 [0.93, 1.08] | 0.978 |  |  |
| During pregnancy, after 28 weeks or during delivery or postnatal | 24 (96.0) | 1.11 [1.02, 1.21] | 0.018 |  |  |
| Current ART regimen* |  |  | 0.466 |  |  |
| First line | 497 (86.4) | Ref |  |  |  |
| 2^nd^/3^rd^ line/unknown | 103 (88.8) | 1.03 [0.96, 1.10] | 0.466 |  |  |
| Missed an ART dose last 7days |  |  | 0.612 |  |  |
| No | 578 (86.9) | Ref |  |  |  |
| Yes | 25 (83.3) | 0.96 [0.81, 1.13] | 0.612 |  |  |
| Facing any ART adherence challenges |  |  | 0.368 |  |  |
| No | 187 (85.0) | Ref |  |  |  |
| Yes | 416 (87.6) | 1.03 [0.97, 1.10] | 0.368 |  |  |

*Variables with missing data, Factors with overall univariate p-value<0.2 bolded, Bolded multivariate significant p-value<0.05. IRR, Incidence rate ratio; CI, Confidence Interval; n=number of cases per category; BMI, Body mass index; ART, Antiretroviral treatment; ANC, Antenatal Care; PVT, Prevention of vertical HIV transmission
